# Image Sensor-Driven 3D Modeling of Complex Biological Surfaces for Preoperative Planning of Hemangioma Treatment

**DOI:** 10.3390/s25185781

**Published:** 2025-09-17

**Authors:** Janis Peksa, Dmytro Kukharenko, Andrii Perekrest, Dmytro Mamchur

**Affiliations:** 1 Information Technology Faculty, Turiba University, Graudu Street 68, LV-1058 Riga, Latvia; janis.peksa@turiba.lv (J.P.); pksg13@gmail.com (A.P.); 2 Institute of Information Technology, Riga Technical University, Kalku Street 1, LV-1658 Riga, Latvia; 3 Computer Engineering and Electronics Department, Kremenchuk Mykhailo Ostrohradskyi National University, Universitetska Street 20, 39600 Kremenchuk, Ukraine; krutoy276@gmail.com

**Keywords:** complex biological systems, hemangioma, preoperative planning, computer models, mathematical modeling, surgical simulation

## Abstract

The advancement of science and technology has elevated the practice of surgery where computer systems now perform the majority of calculations required for successful interventions. This technological progress can be leveraged to foster surgical improvements by developing and implementing novel computer models for the preoperative planning of surgical treatments. Such systems enable surgeons to select optimal treatment tactics and dosages of operative interventions tailored to individual patients. Currently, there is no consensus on the use of expectant management for hemangiomas, as the most effective therapeutic strategy often depends on the tumor’s type and location, with early treatment being critical in some cases. Accurate diagnosis and effective treatment necessitate precise determination of the tumor’s type, growth characteristics, structure, and location. The use of a surgical method for hemangiomas removal is better for the removal of small formations in places that are not critical from a cosmetic prospective (for example, for males this might be the back and legs). This paper presents a method for creating a three-dimensional (3D) model of hemangioma using polynomial approximation and spline modeling to assist surgeons. The development of the mathematical model, the software implementation, and a comprehensive error analysis are explained in this work. The resulting model demonstrated an average approximation error of 5.6%, and a discriminant analysis confirmed the significance of five key parameters for successful resection. The proposed system offers a robust and economically viable tool for improving the accuracy and outcomes of hemangioma surgery.

## 1. Introduction

With the modern development of science and technology, surgery is advancing to a new level, largely due to computer systems that perform the complex calculations necessary for successful surgical interventions [1,2,3,4,5,6,7,8,9,10,11,12]. To support surgeons, it is essential to develop and implement new computer systems and models for preoperative planning [2,3,4]. Computer modeling of complex biological systems uses a variety of methods and tools to study the structure, dynamics, and functions of living organisms and their components. Using such systems, surgeons can determine the optimal tactics and extent of surgical interventions for each patient, a concept that has evolved significantly in recent decades [13,14].

Hemangiomas are the most common benign vascular tumors in children, affecting up to 10% of infants and accounting for up to 50% of all neoplasms in this demographic [5,7,15]. Although clinically considered non-malignant, hemangiomas are characterized by rapid, progressive growth that can destroy surrounding tissues and cause significant cosmetic and functional damage [8,9,16]. While diagnosing external hemangiomas is straightforward, the key question of whether to treat and how to treat remains unsolved [5,6]. The debate continues over immediate intervention versus a “wait-and-see” approach [17]. Current combination treatments do not always yield excellent results, necessitating research into new methods that utilize innovative technologies [18]. A successful outcome must achieve excellent oncological, functional and cosmetic results.

The surgical removal of hemangiomas is often the best solution for small formations in non-critical cosmetic areas (for example, for men, this could be the back, the legs, etc.). Such a procedure is typically performed under general anesthesia, where the tumor and a few millimeters of healthy tissue are removed. For deeply grown hemangiomas, the decision for surgery depends on its size and depth [19,20]. Pre-surgical measures, such as medication or radiation, are sometimes used to reduce tumor size.

An analysis was conducted on the structure, etiology, and management of human hemangiomas. Infantile hemangioma is the most prevalent tumor in the neonatal period. Its incidence varies: approximately 1.1–2.6% of neonates are affected according to [21], rising to 4–10% by 12 months of age. There is a strong female predominance, with a female-to-male ratio of about 2–3:1 (some reports suggesting 3:1–5:1). Clinical presentation typically occurs shortly after birth. About 30% of hemangiomas are present at birth, while the remaining approximately 70% emerge within the first month of life. When a neonate presents with one hemangioma, there is 75% likelihood of developing additional during the first six months.

Currently, a number of hemangioma treatment methods exist [10,11]. Unfortunately, they do not always help, so surgical intervention is inevitable. Planning a high-quality surgical intervention for hemangioma treatment is extremely difficult because existing methods are based on the averaged statistical data and fail to account for individual anatomical variations [4]. Computer planning technologies, including virtual modeling, can significantly simplify a doctor’s work, help to avoid errors and prepare surgeons for unforeseen complications [22,23,24,25]. The primary goal of computer planning is to select the least traumatic surgical approach for a patient based on their specific anatomy. This research was conducted to develop simulation tools to assist surgeons in planning hemangioma treatment.

## 2. Materials and Methods

### 2.1. Mathematical Description of Biological Surface

**Figure 1:**
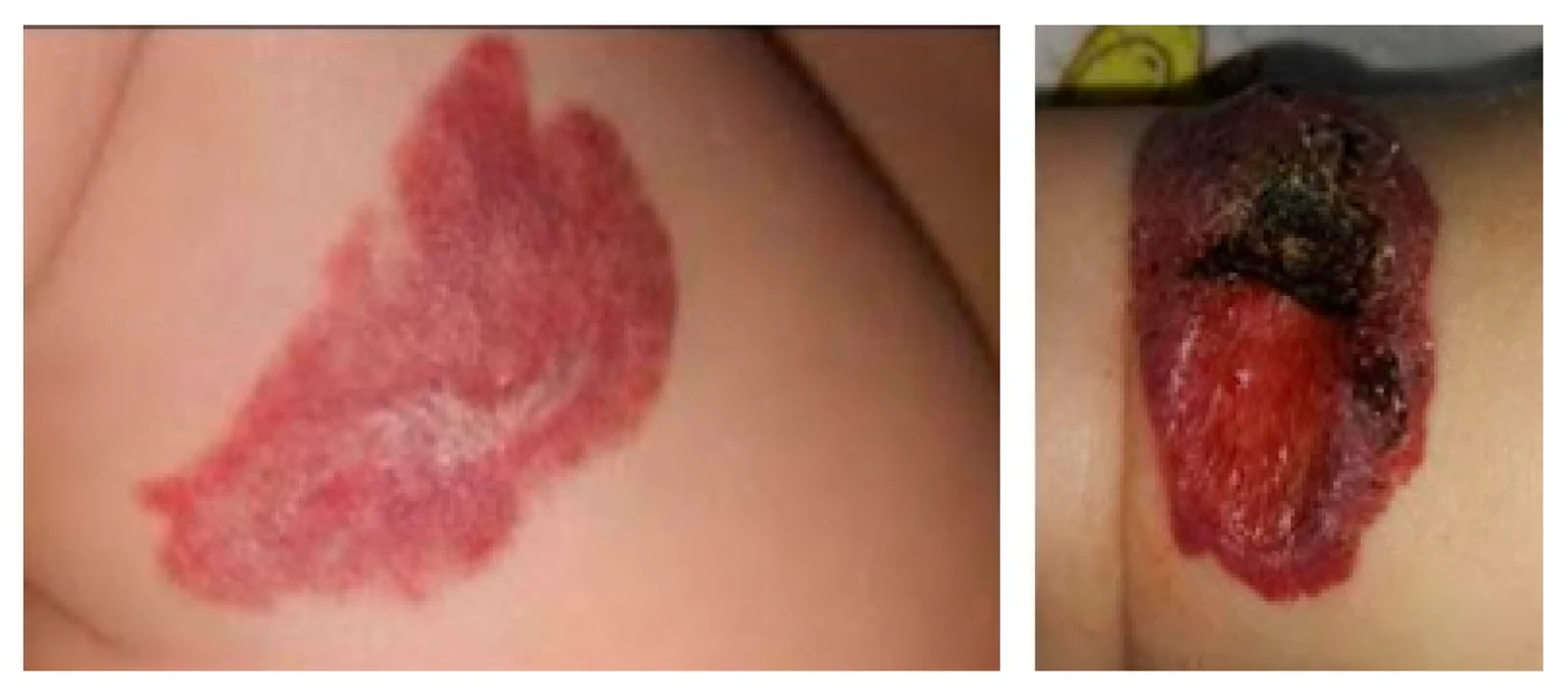
Hemangiomas with an irregular shape and volume.

**Figure 2:**
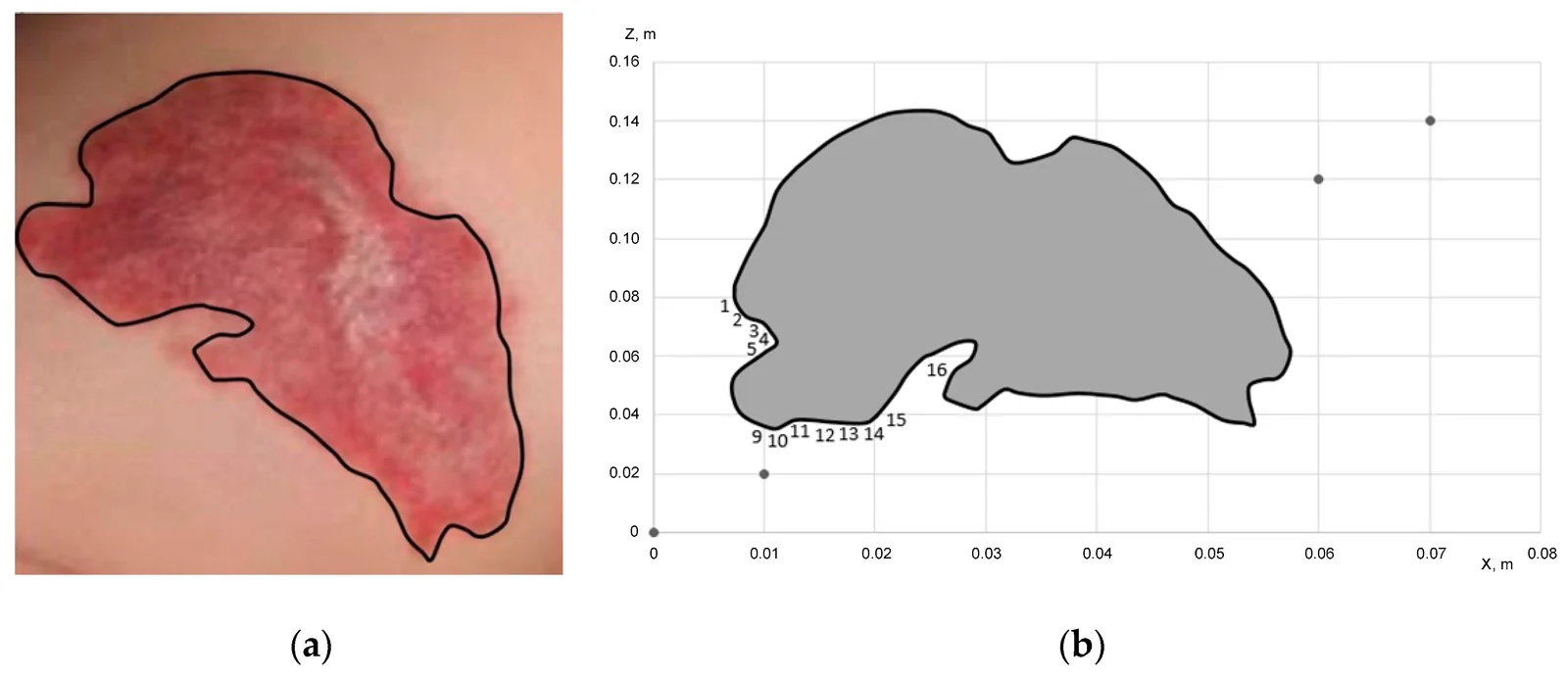
Modeling of hemangioma: (**a**) construction of a two-dimensional model of hemangioma based on the provided images; (**b**) projection of the hemangioma model onto the ZOX plane.

Traditional methods for determining the need for treatment often assume a rounded shape for hemangiomas, which is rarely the case in reality (Figure 1) [12]. In our previous work [10,11] the irregular boundary of the hemangioma (Figure 2a) was projected onto a 2D plane (Figure 2b) and partitioned into distinct segments based on curvature. Specifically, the concave region of the contour, labeled as segment 1–5 in Figure 2b, was approximated using a third-degree polynomial (1), while the more complex convex region, labeled as segment 9–16, was modeled with a fifth-degree polynomial (2). This piecewise approach allowed for a more precise fit to the non-uniform shape.

In the method of determining the indications for the treatment of hemangiomas in children [12], it is believed that hemangioma has a rounded shape. But this is not always the case (Figure 1). In general, hemangioma always has an irregular shape. Based on the results presented in previous works [10,11], the periphery of the hemangioma shown in Figure 1, left-hand side image, was partially approximated (concave part, points 1–5 Figure 2b and convex part, points 9–16) by polynomials of the third and fifth degrees, presented in Equations (1) and (2), respectively.

Z = (896.436 × 3 − 64.994 × 2 + 1.206x + 0.023) + G;(1)

Z = (9617.999 × 5 − 847.094 × 4 − 104.9966 × 3 +15.369 × 2 −0.661x + 0.011) + G, (2)

where G is an additional correction factor.

For irregular shapes, the size and area of the hemangioma are determined approximately [12] typically, using a transparent film marked with squares, by placing the film on the hemangioma and estimating the area of the hemangioma by the sum of the squares and half-squares covering the hemangioma.

To more accurately determine the area and volume of irregularly shaped hemangiomas, we propose a method using computer technology. From a photograph, the hemangioma’s boundaries can be outlined using image processing software [26,27]. By applying a mesh of triangular elements within these boundaries, we can calculate not only its area, but also its volume and construct 3D model [28]. This model, combined with patient-specific metabolic parameters, allows for predicting the dynamics of hemangioma growth over time. To construct a refined model, we use the following equations to determine the volume (V), mass (m), mean density (μ), and center of mass ($x_{mean}$, $y_{mean}$, $z_{mean}$) in Cartesian and cylindrical coordinates. This requires establishing a reliable coordinate system, a challenge discussed in broader neuroimaging contests [29,30,31].

In this work, a method for determining the area of the hemangioma using triangular elements is proposed. Let us assume, a patient M. has a hemangioma (Figure 2a). It consists of 41,127 calculated triangular elements (linear element of the size 4 × 10^−4^ m, providing a resolution of 0.4 mm).

To construct a refined model of hemangioma, we will use the following equations, for example, determining the volume of hemangioma (3, 7), its mass (4, 8) through Cartesian and cylindrical coordinates and densities (5), as well as the coordinates of its center (6):

(3) $$V=\underset{G}{∭}dxdydz,$$

(4) $$m=\underset{G}{∭}\mu (x,y,z)dxdydz,$$

(5) $$\mu_{mean}\left(x,y,z\right)=\frac{m}{V}=\frac{\underset{G}{∭}\mu (x,y,z)dxdydz}{\underset{G}{∭}dxdydz},$$

(6) $$x_{mean}=\frac{1}{m}=\underset{G}{∭}x\mu dxdydz,\;y_{mean}=\frac{1}{m}=\underset{G}{∭}y\mu dxdydz,\;z_{mean}=\frac{1}{m}=\underset{G}{∭}z\mu dxdydz,$$

(7) $$V=\underset{G}{∭}dxdydz=\underset{G}{∭}rdrd\varphi dz,$$

(8) $$m=\underset{G}{∭}\mu (x,y,z)dxdydz=\underset{G}{∭}\mu^{\prime }(r\;cos\varphi ,\;r\;sin\varphi ,\;z)rdrd\varphi dz,$$

Here, $\mu_{mean}\left(x,y,z\right)$ represents the physical density of the hemangioma tissue at a given spatial coordinate (in units such as g/mm^3^), and is distinct from any pixel intensity or color values from the input images. For the purpose of mass estimation, this can be approximated as a uniform value based on typical biological tissue densities or informed by other imaging modalities.

Furthermore, when determining the surface area of a hemangioma, S, the biomechanical properties of the overlying skin must be considered. The skin is stretched by the underlying tumor mass, and this tension affects the true surface area. We account for this using the following conceptual relationship:

(9) S=k×∫dS,

where ∫dS is the geometrically calculated surface area and k is a dimensionless skin elasticity coefficient. This coefficient accounts for age-dependent changes in skin elasticity; as skin ages, its elasticity modulus changes, making it stiffer and less extensible. Taking this into account is crucial for accurate surgical planning, particularly for estimating the tissue requirements for wound closure after excision.

### 2.2. 3D Surface Reconstruction from 2D Images

**Figure 3:**
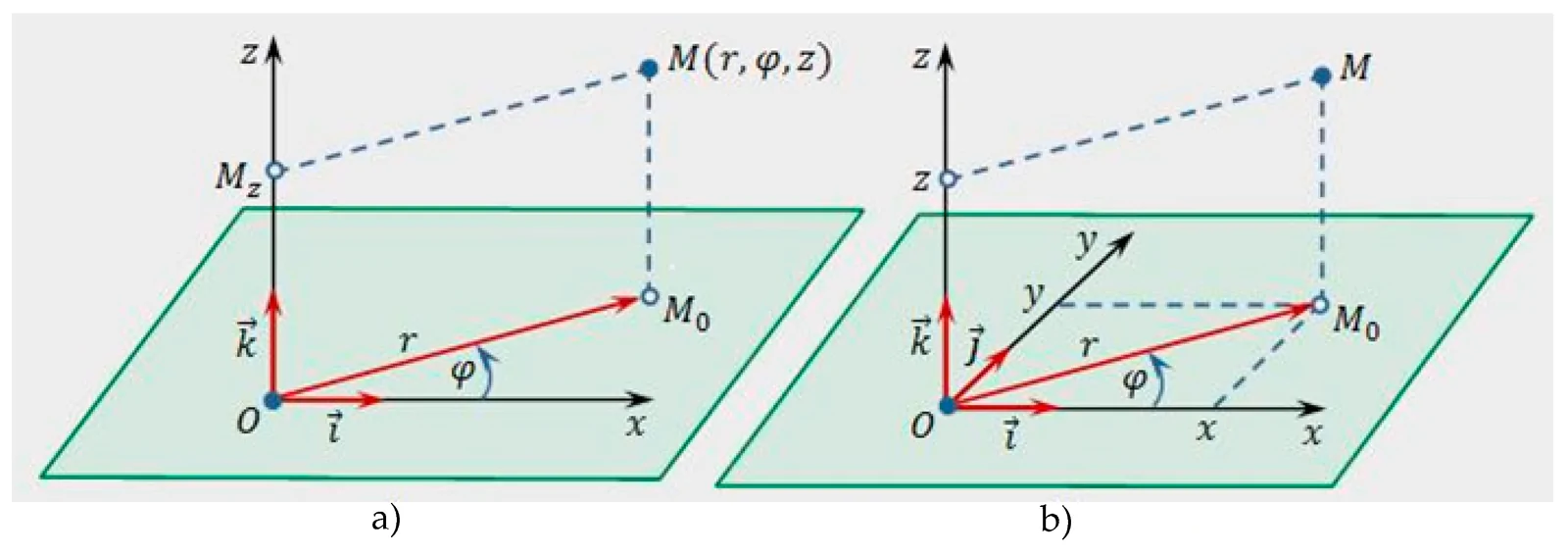
Method of determining coordinates on the surface of the hemangioma model: (**a**) cylindrical coordinate system; (**b**) Cartesian coordinate system.

The method of determining the coordinates on the surface of the hemangioma model when point M passes into point M_0_ is visualized in Figure 3 and new coordinates recalculation is described by Equations (10) and (11).

**Figure 4:**
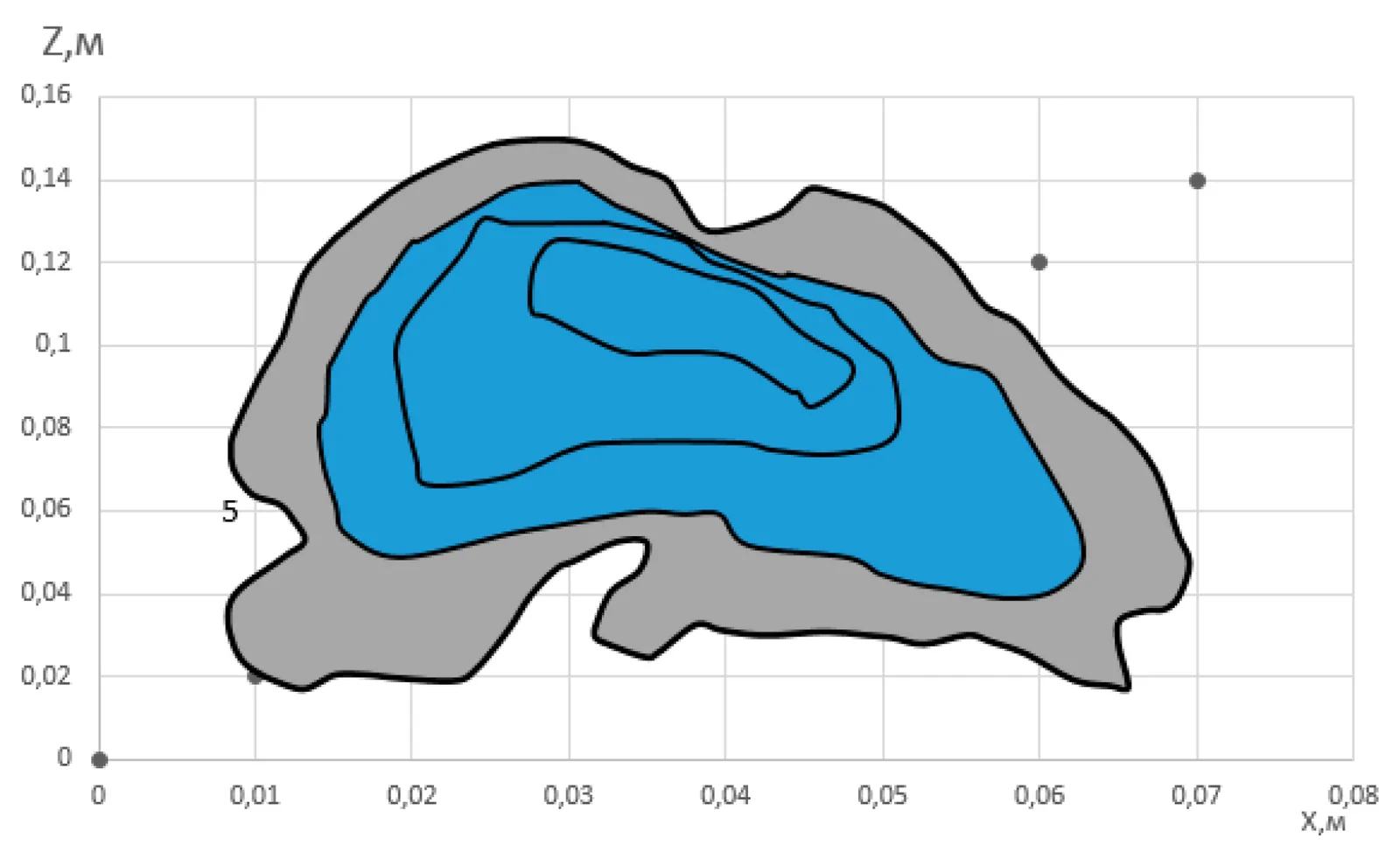
Three-dimensional image of hemangioma.

An image of derived hemangioma representation in space is shown in Figure 4.

(10) $$\left\{\begin{array}{c} x=r\;cos\varphi ;\\ y=r\;sin\varphi ;\\ z=z. \end{array}\right.$$

(11) $$\left\{\begin{array}{c} r=\sqrt{x^{2}+y^{2}};\\ \cos\varphi =x/r=\frac{x}{\sqrt{x^{2}+y^{2}}};\;\\ \sin\varphi =y/r=\frac{y}{\sqrt{x^{2}+y^{2}}};\\ z=z. \end{array}\right.$$

where the angle φ is calculated as φ=atan(y, x), where −π≤φ≤π

Let us consider a regular surface in parametric form. A regular surface is a set of points M(x, y, z) of 3-dimensional space, the coordinates x, y, z of which are determined from the following relations:

(12) $$x=x\left(u,\;v\right),\;y=y\left(u,\;v\right),\;z=z\left(u,\;v\right),\;\left(u,\;v\right)\in D\;,$$

where x(u, v), y(u, v), z(u, v), are smooth functions of their arguments, and the following relation holds:

(13) $$rank\left[\begin{array}{ccc} x_{u}(u,\;v) & y(u,\;v) & z(u,\;v)\\ x_{v}(u,\;v) & y_{v}(u,\;v) & z_{v}(u,\;v) \end{array}\right]=2.$$

The term ‘rank’ refers to the number of linearly independent column (or row) vectors in the matrix. This condition ensures that the tangent vectors ${\vec{r}}_{u}$ and ${\vec{r}}_{v}$ are linearly independent at every point, which guarantees the existence of a unique, non-degenerate tangent plane to the surface at that point.

The domain D is some region on the plane of parameters u, v. The equations given above are called parametric equations of the surface. They are often written in vector form:

(14) $$\vec{r}=\vec{r}\left(u,\;v\right),\;\;\left(u,\;v\right)\in D\;.$$

In this case, the point M lies on the surface and will be defined by two parameters M(u, v). For example, consider the surface of a sphere with the radius R. The equation of the sphere has the following form:

(15) $$x^{2}+y^{2}+z^{2}=R^{2}.$$

It is convenient to choose spherical coordinates u=φ, v=Θ as parameters. Then the parametric equations will take the following form:

(16) $$\left\{\begin{array}{c} x=R\cos u\cos v;\\ y=R\cos u\cos v;\\ z=R\sin u. \end{array}\right.$$

Let the region of change in parameters u, v be the following region D:

(17) $$D=\left(0\leq u\leq \frac{\pi }{2},\;\;\frac{\pi }{12}\leq v\leq \frac{5\pi }{12}\right).$$

It should be noted that this spherical example and its specific parameter limits are provided for illustrative purposes only to explain the concept of a parametric surface. They were not used in the reconstruction of the hemangioma model, where the parameter domains are defined by the physical extent of the captured 2D cross-sections.

In the region D, there are also two lines u = const and v = const. Using these lines, a coordinate grid can be constructed in the region D. This grid can then be mapped onto an element of the spherical surface using proper equations. If we calculate the partial derivatives with respect to the parameters (u, v) of the radius vector r(u, v), we can find the tangent vectors to the surface under consideration.

(18) $$\vec{r_{u}}\left(u,\;v\right)=\left(\frac{\partial }{\partial u}x\left(u,\;v\right),\;\;\frac{\partial }{\partial u}y\left(u,\;v\right),\;\;\frac{\partial }{\partial u}z\left(u,\;v\right)\right),$$

(19) $$\vec{r_{v}}\left(u,\;v\right)=\left(\frac{\partial }{\partial v}x\left(u,\;v\right),\;\;\frac{\partial }{\partial v}y\left(u,\;v\right),\;\;\frac{\partial }{\partial v}z\left(u,\;v\right)\right).$$

The normal vector can be found as the cross product of the tangent vectors:

(20) $$\vec{N}={\vec{r}}_{u}\times {\vec{r}}_{v}.$$

To find the projections of the normal vector, it is convenient to write the cross product in determinant form:

(21) $$\vec{N}=\left|\begin{array}{ccc} \vec{i} & \vec{j} & \vec{k}\\ x_{u} & y_{u} & z_{u}\\ x_{v} & y_{v} & z_{v} \end{array}\right|.$$

The condition in the definition of a regular surface means that at each point M of a regular surface there is a tangent plane, and this plane changes continuously as the current point M moves along the surface. The tangent vectors and the normal vector change continuously. The equation of the tangent plane passing through the point M belonging to this surface will have the following form. Let the coordinates of this point be equal to M(x_0_, y_0_, z_0_). Let the normal vector N to the surface at this point also is known. Then the equation of the tangent plane will have the following form:

(22) $$N_{x}\left(x-x_{0}\right)+N_{y}\left(y-y_{0}\right)+N_{z}(z-z_{0})=0.$$

A plane is a 2-dimensional object, so it can, like any surface, be described by two parameters using parametric equations.

If the figure does not have sharp bends and depressions, then its three-dimensional shape can be recreated using radial sections. The corresponding sections can be obtained by taking photographs when rotating the figure at fixed angles. Depending on the optical properties (e.g., surface reflectivity and color) of the hemangioma, it is desirable to photograph the section on a contrasting background: a light background with a dark surface of the figure, or a dark background with the foreground of the figure illuminated. This will allow binary image processing to be applied to highlight the contour of the figure and determine the coordinates of the radial section. As a result, we have a set of data in a combined coordinate system for the specified angles of section θ: z=f(r,θ). The input data is a polar coordinate system in the plane of the base *xy* of the figure (r,θ), and the output data is the height of the figure *z*. If the figure has an axis of symmetry, then it is convenient to place it at the origin of the plane *xy*. Therefore, the discrete coordinates *r* will have both positive and negative values relative to the axis of symmetry of the figure.

The quality of the 3D reconstruction is fundamentally dependent on the characteristics of the image acquisition system. For this application, desirable camera features include high spatial resolution to resolve fine surface details, high bit depth (e.g., 12-bit or 14-bit) to capture subtle gradations in color and intensity, and excellent color fidelity to ensure accurate clinical representation. Furthermore, the use of standardized, diffuse lighting is critical to minimize specular reflections and hard shadows, which can be misinterpreted by reconstruction algorithms and introduce surface artifacts.

### 2.3. Computational Model Development and Software Implementation

**Figure 5:**
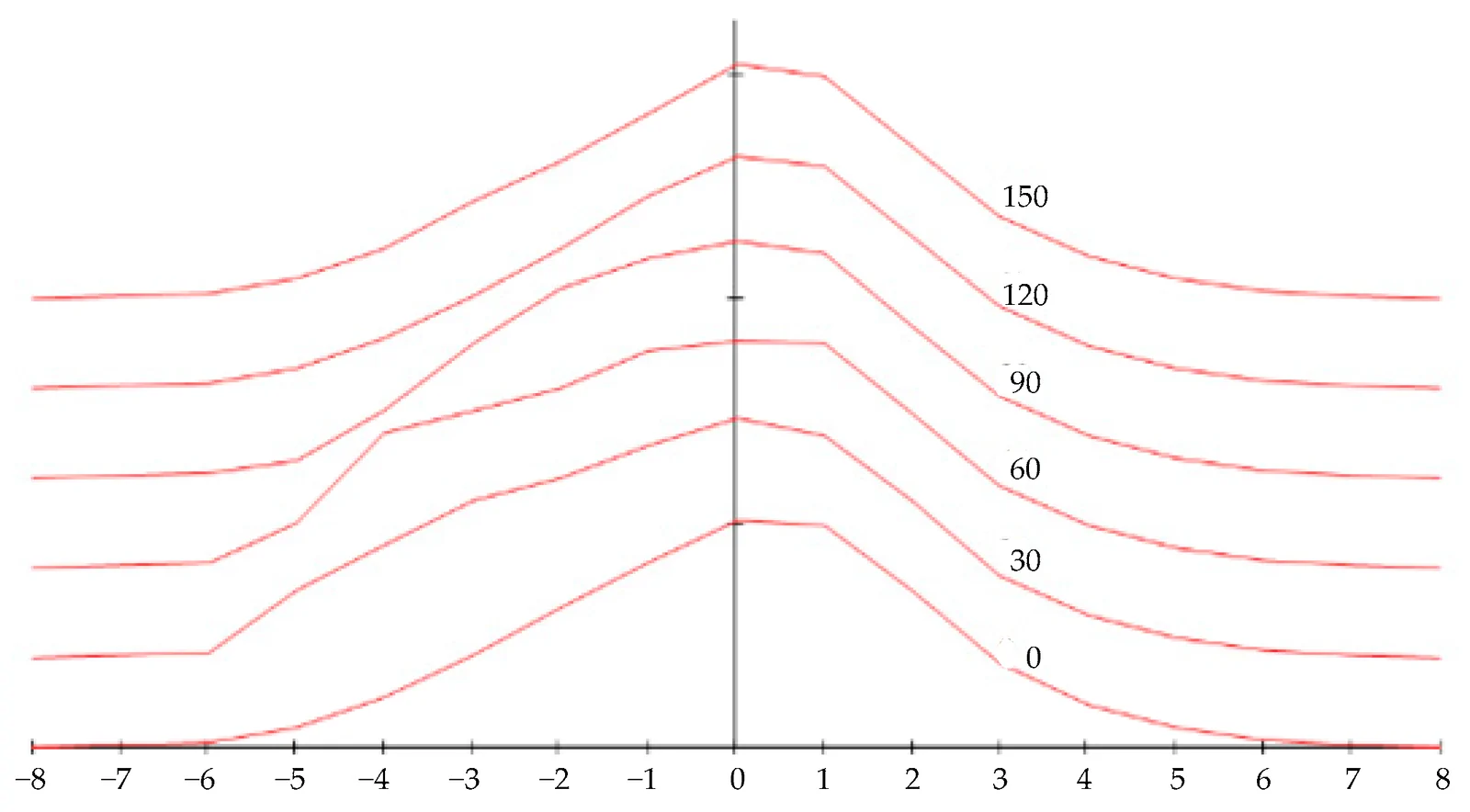
Surface cross-section graphs for modeling.

To develop and validate the simulation model, a physical phantom with a complex surface analogous to a hemangioma was created. This phantom was rotated at 30° intervals and photographed to generate a set of six 2D cross-sections. The graphical representations of these cross-sections, shown in Figure 5, served as the input data for the software workflow described below. This phantom-based approach allowed for a controlled evaluation of the reconstruction algorithm. The clinical case shown in Figure 2 serves as an example of the type of input image the finalized system is designed to process.

**Figure 6:**
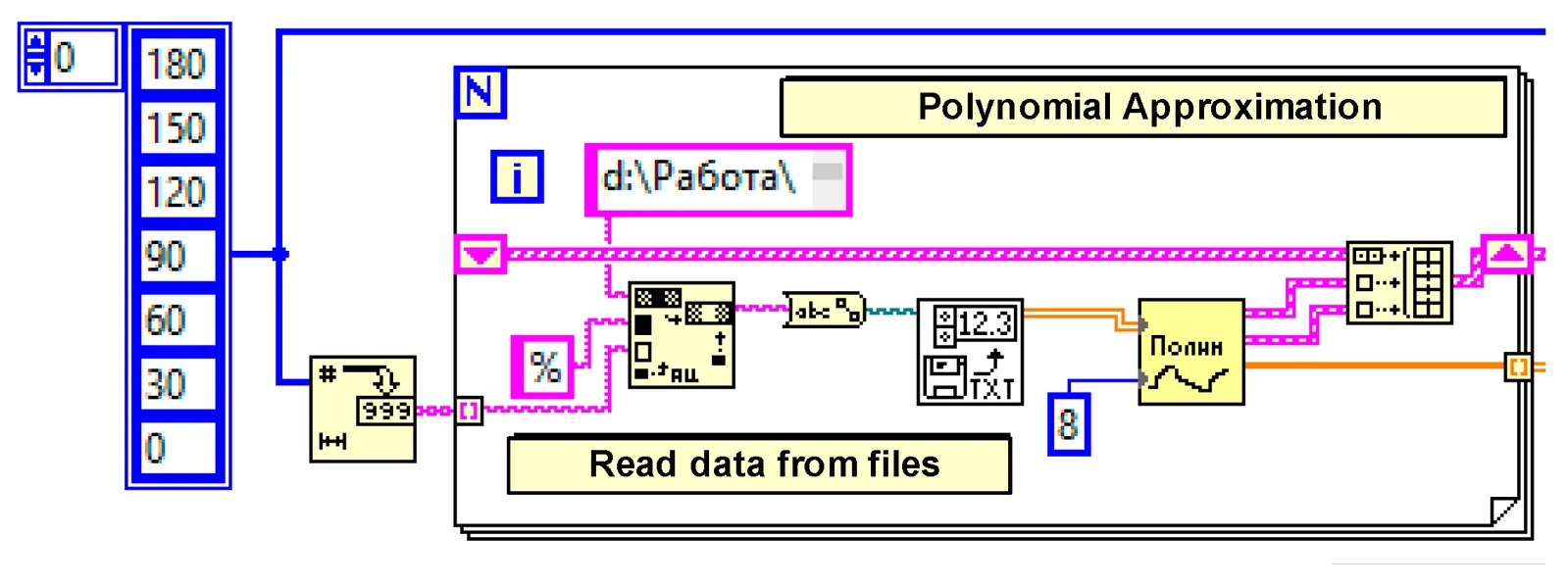
Block diagram of input data processing.

**Figure 7:**
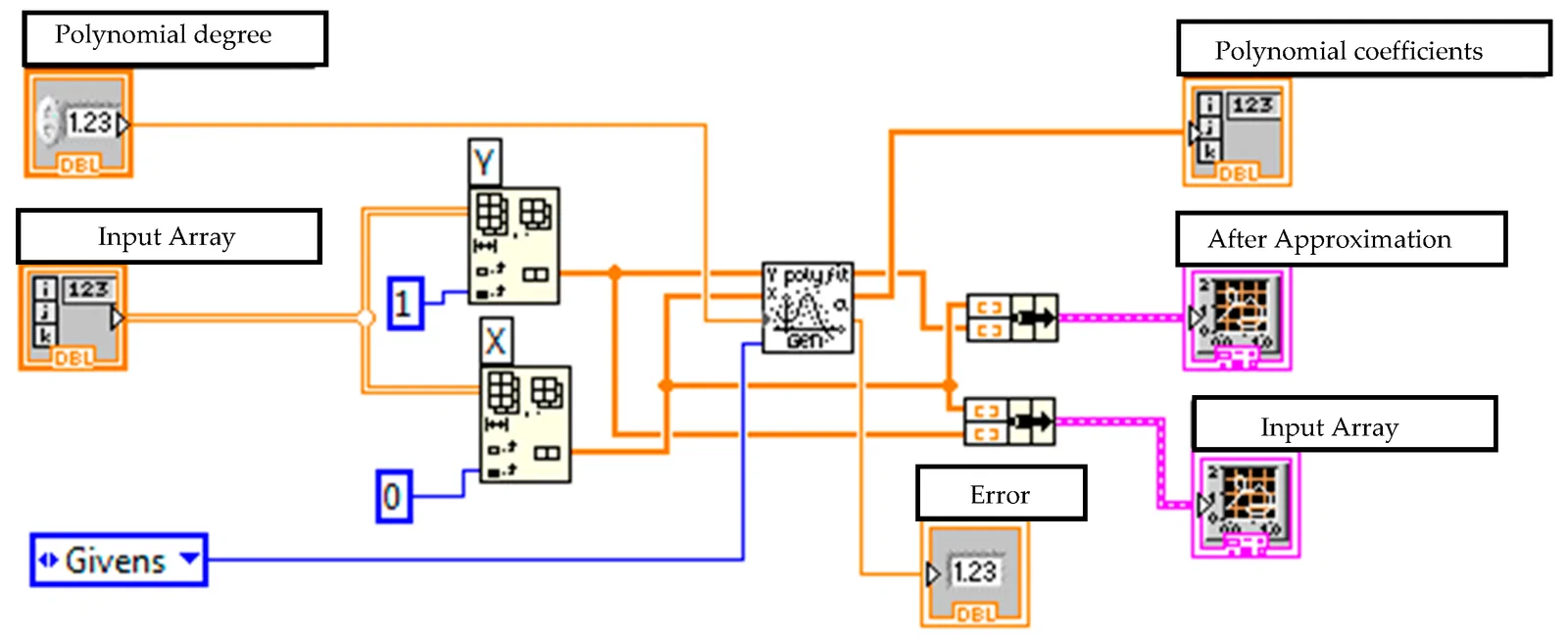
Virtual device for performing polynomial approximation of data and displaying the results.

**Figure 8:**
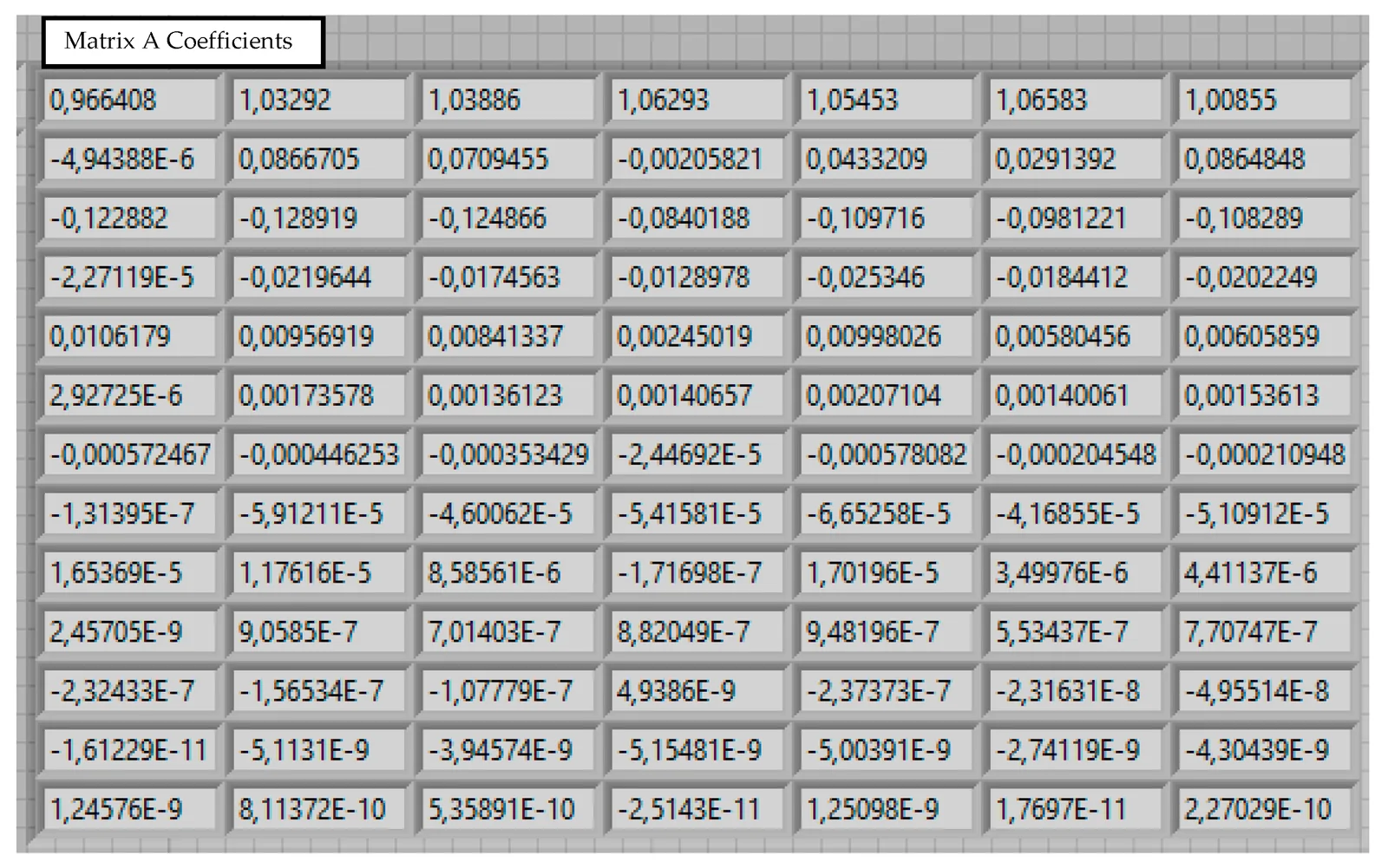
Calculated coefficients for matrix *A*.

**Figure 9:**
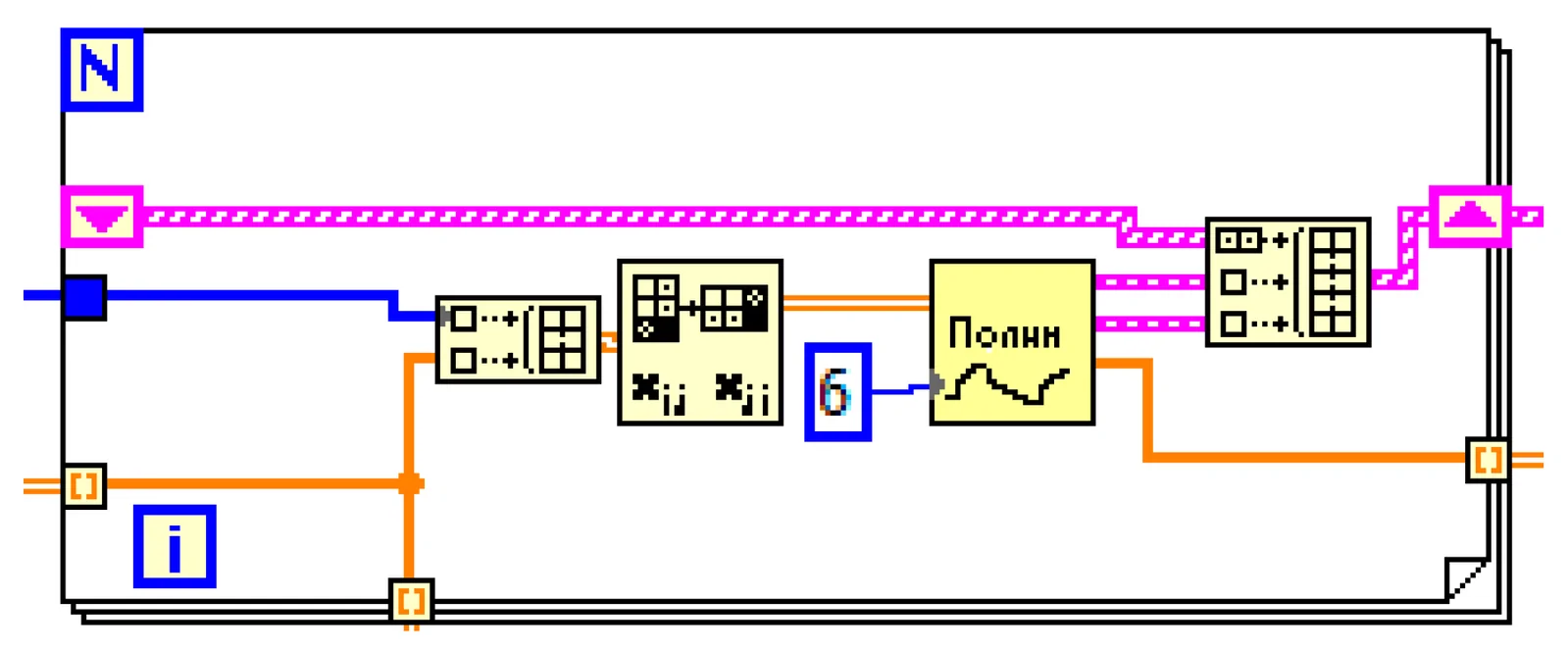
Virtual device for approximating coefficients *a*.

**Figure 10:**
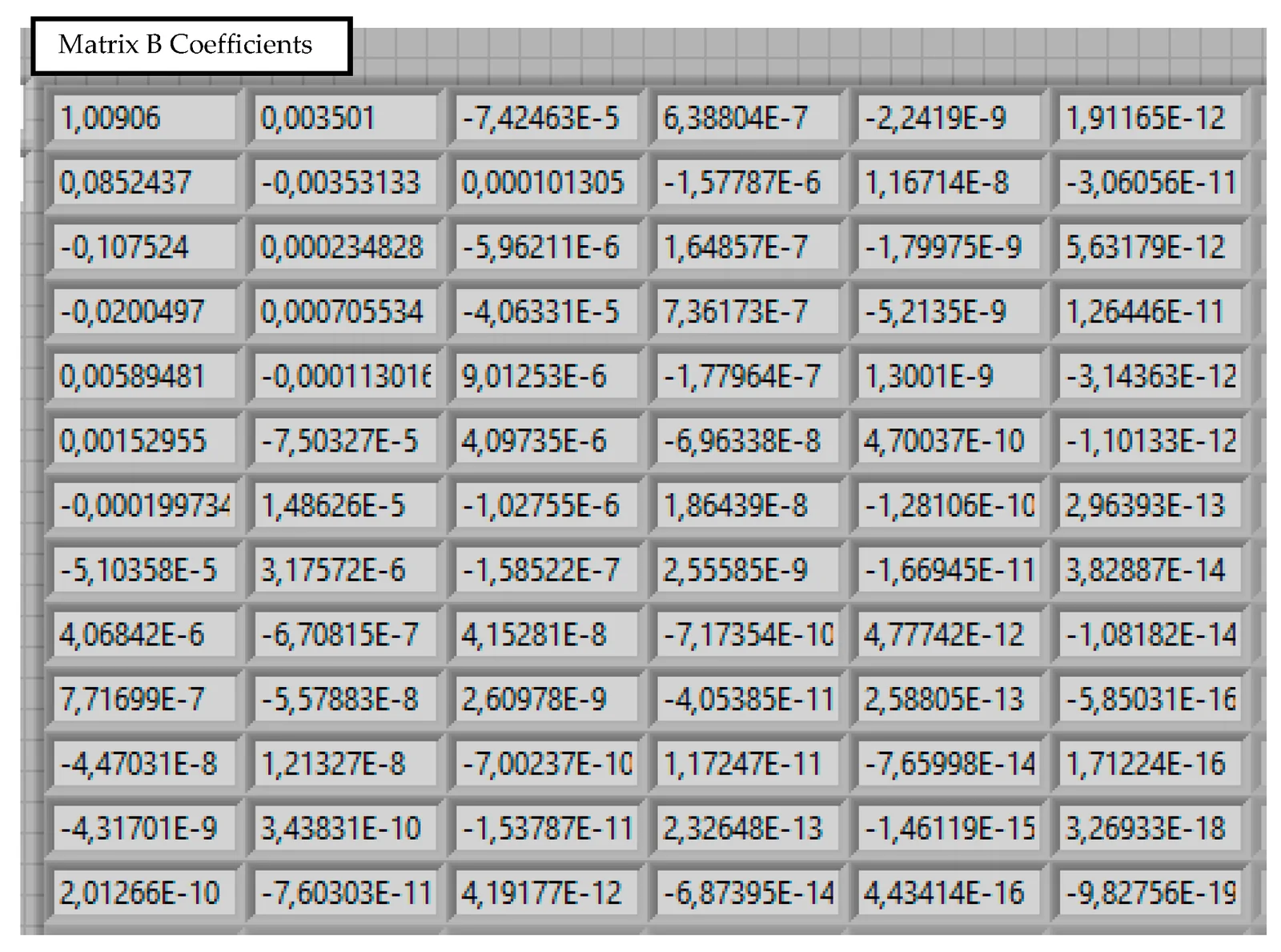
Calculated coefficients for B matrix.

**Figure 11:**
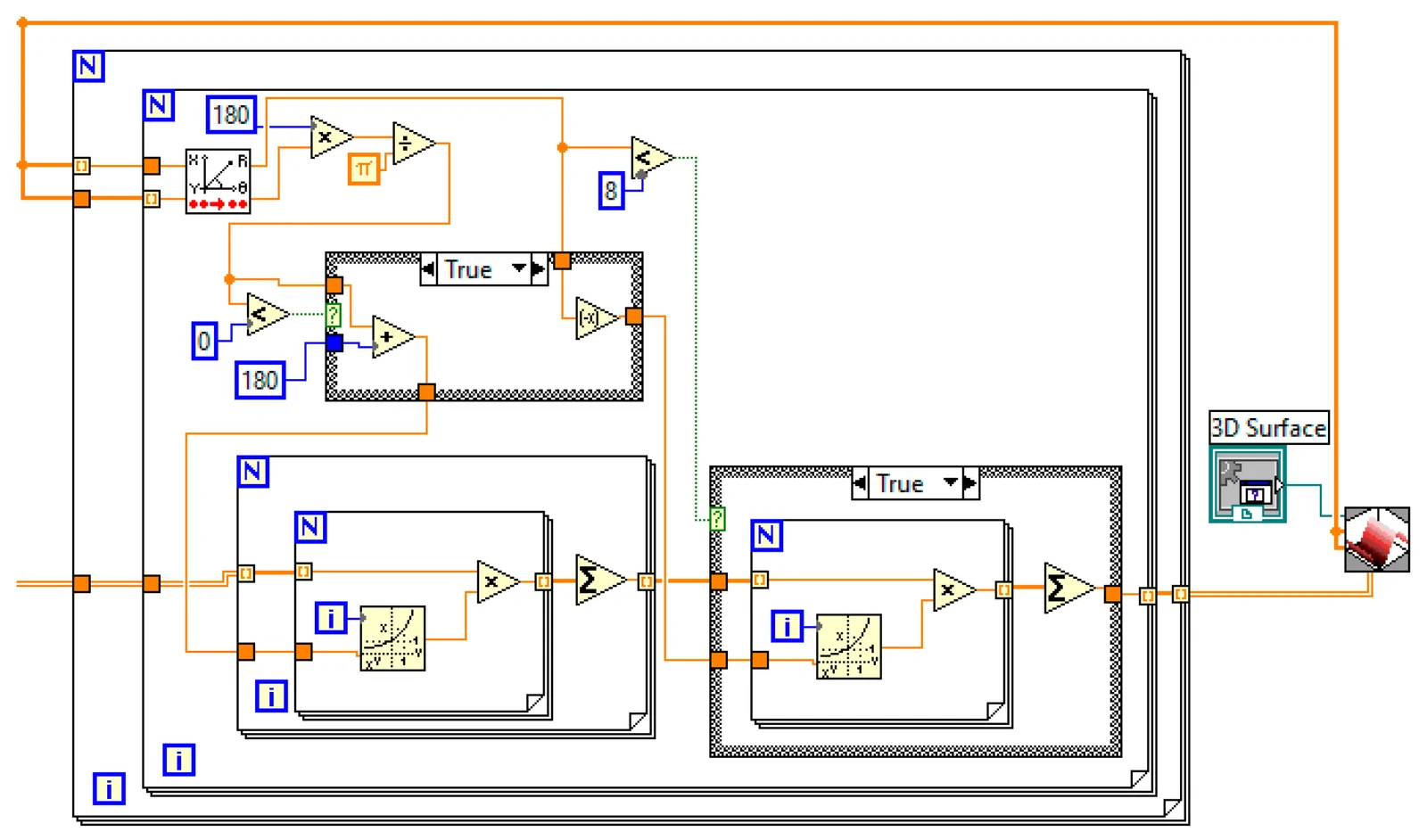
A fragment of a program for converting input polar coordinates for intersections to a Cartesian system.

The LabVIEW 2021 software (National Instruments, Austin, TX, USA) was used for data processing and model development. LabVIEW is a graphical programming environment where programs, known as Virtual Instruments (VIs), are created. Each VI consists of a front panel, which serves as the user interface, and a block diagram, where graphical icons representing functions are connected by wires to control the flow of data. This dataflow paradigm is highly suited for applications involving measurement, automation, and signal processing. The block diagrams and screenshots shown in Figure 6, Figure 7, Figure 8, Figure 9, Figure 10 and Figure 11 represent the data processing logic implemented in our system.

The input data, representing the surface height at different sections (Figure 6), are read from separate files as numerical values.

The output data representing height coordinates z_i_ relative to x_i_ are sequentially read from separate files, the names of which coincide with the corresponding intersections y_k_:

(23) $$z_{k}\left(x\right)=\sum_{i=0}^{N}a_{i}x^{i}.$$

These data are then approximated using polynomial dependencies to achieve the best fit. A general polynomial is performed using the *General Polynomial Fit VI*, a standard function within the LabVIEW analysis library. This VI takes arrays of independent (X) and dependent (Y) data, along with a specified polynomial order, and returns the set of polynomial coefficients that best fits the data using a least-squares method. This VI was used to obtain the polynomial coefficients ($a_{i}$) for chosen polynomial order (N = 13). The choice of a 13th-order polynomial was determined empirically by evaluating a range of orders; this value was found to provide the optimal balance between accurately fitting the complex cross-sectional contour of our phantom model and avoiding the instability and overfitting that can occur with excessively high-order polynomials. A block diagram implementing polynomial approximation presented in Figure 7.

The executive elements of the listed operations are combined into a custom virtual device implementing polynomial approximation. The result is a two-dimensional matrix A, where each row *k* contains the coefficients а_k0_ … а_kn_, corresponding to a specific intersection *y*:

(24) $$A=\left(\begin{array}{cccc} a_{00} & a_{01} & \cdots & a_{0N}\\ a_{10} & a_{11} & \cdots & a_{1N}\\ \vdots & \vdots & \ddots & \vdots \\ a_{M0} & a_{M1} & \cdots & a_{MN} \end{array}\right).$$

The results of calculating the coefficients are shown in Figure 8.

Each column *i* of this matrix corresponds to a coefficient *a_i_* at different cross-section coordinates. This allows for establishing a functional dependence through another layer of polynomial approximation:

(25) $$a_{i}\left(y\right)=\sum_{k=0}^{M}b_{ik}y^{k}.$$

The specified action is implemented by the corresponding cyclic structure shown in Figure 9.

The final result is a matrix of coefficients B (Figure 10), which allows for the reconstruction of the surface height *z* for any given *x* and *y* coordinates (26). This spline-based approach is effective for reconstructing complex 3D shapes from 2D data [32].

The result of the calculation is a two-dimensional matrix of coefficients $b_{ik}$ (Figure 10): the *i*-th row $b_{i1}{\ldots b}_{iM}$ is used to calculate the coefficient $a_{i}$. Therefore, the obtained matrix of coefficients allows us to reproduce the surface *z* for arbitrary values of the parameters *x* and *y*:

(26) $$z\left(x,y\right)=\sum_{i=0}^{N}\left(\left(\sum_{k=0}^{M}b_{ik}y^{k}\right)x^{i}\right).$$

In our case, M = 5, N = 12.

This dependence is implemented in LabVIEW environment and is shown in Figure 11. To be able to use the obtained data in other programs using additional software, the coefficient matrix is imported into an external data file.

## 3. Results

### 3.1. Three-Dimensional Model Construction and Visualization

**Figure 12:**
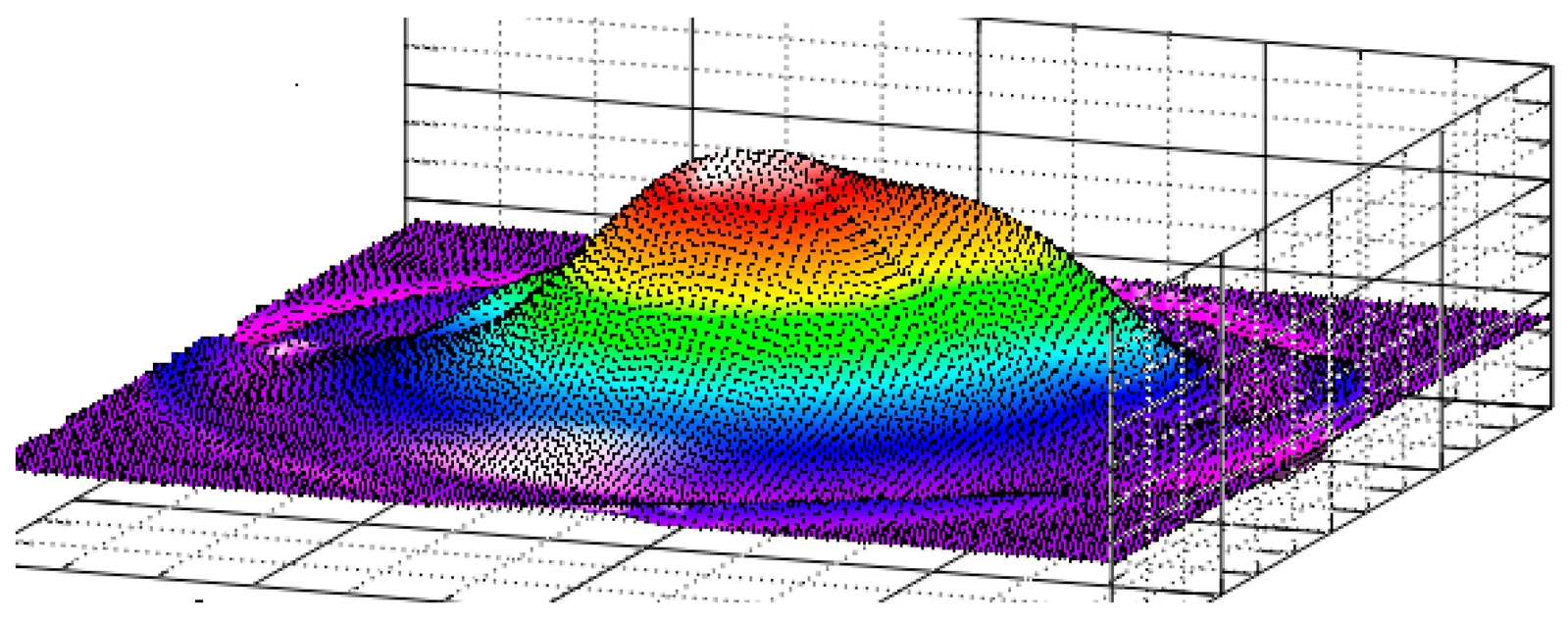
Reproduced 3D surface of a figure in a Cartesian coordinate system using the equations of radial sections.

The final stage of modeling is the construction of the 3D surface (Figure 12). Developed method allows for the recreation of a complex surface from a series of 2D images using polynomial approximation, which is less computationally intensive than other known 3D modeling techniques used for this purpose [33,34]. The accuracy can be enhanced by increasing the number of radial section or the order of the polynomials. This is analogous to how computer tomography slice thickness affects 3D reconstruction quality [19,35].

As a result, based on frontal images of the object, taken from various angles forming wide viewing sector, a method for reconstructing a 3D surface using polynomial approximation is proposed. Specifically, a special case of the Lagrange polynomial is employed, as it allows for efficient modeling of complex surfaces with reduced computational effort compared to conventional 3D modeling techniques. In this approach, the surface is represented by a matrix of coefficients with dimensions 9 × 6 or 13 × 6. If higher accuracy is required, it can be achieved by increasing the number of terms in the polynomial series and the number of radial sections used in the reconstruction.

We also developed software implementing Non-Uniform Rational B-Splines (NURBS) to create smooth, organic shapes ideal for biological modeling [25]. Unlike polygon-based models, NURBS surfaces are inherently smooth and can be scaled without loss of detail, making them superior for this application.

**Figure 13:**
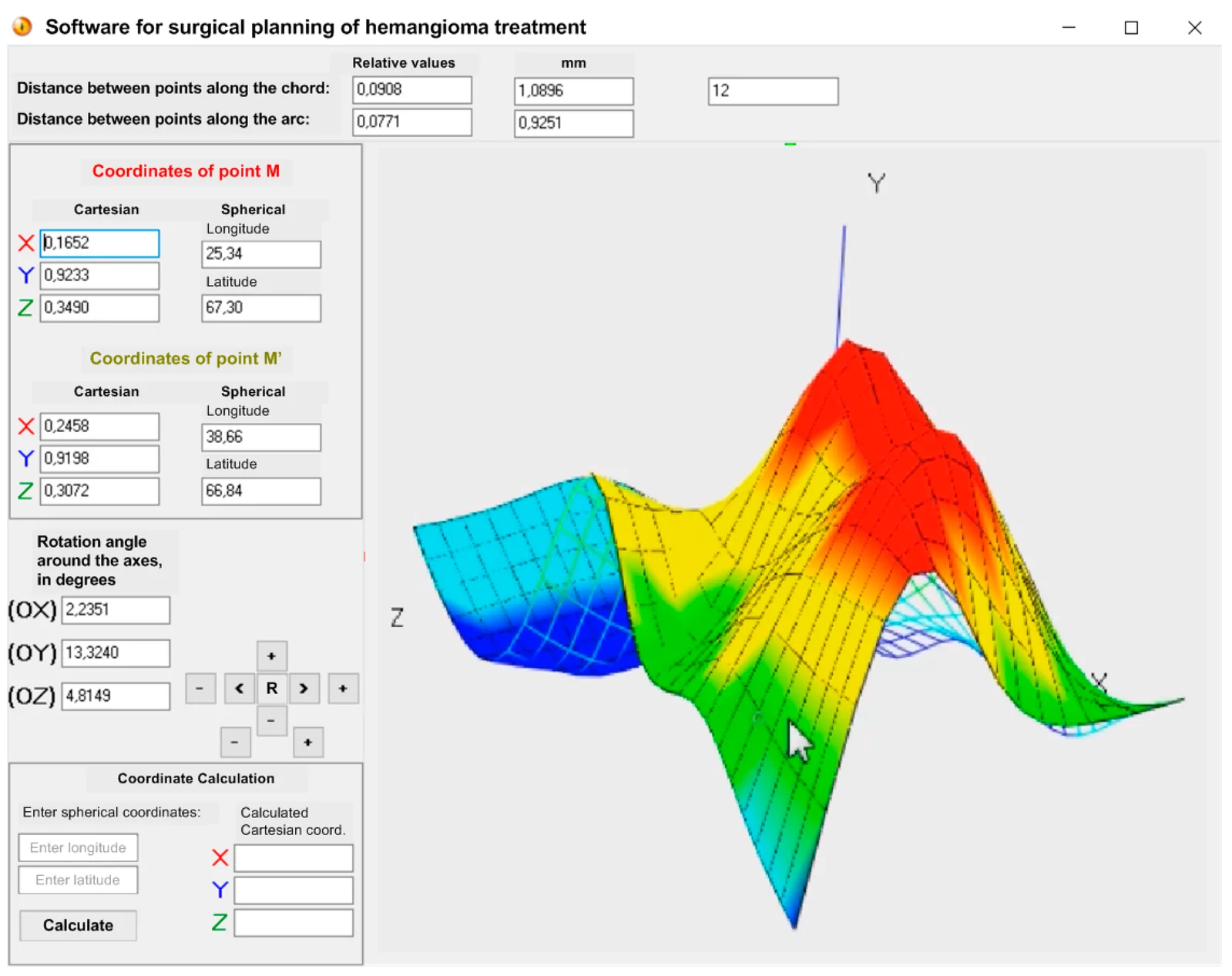
Working window of the proposed software featuring three-dimensional model of hemangioma.

The software receives input data from a medical camera to generate a spatial model of the hemangioma. An operator can then place two arbitrary points on the model to calculate the required resection size and assess the tumor’s depth based on its topography (Figure 13). The use of such models in clinical practice is growing, particularly for planning complex interventions with technologies like interoperative MRI and electromagnetic navigation [36,37,38,39].

In developing a three-dimensional qualitative model of a hemangioma, spline modeling was chosen over polygonal modeling due to its higher accuracy and scalability. Unlike polygonal models, spline-based models maintain consistent quality during scaling and approximation. In spline modeling, the shape of the hemangioma is defined by a set of curves distributed along the equator of a reference sphere. This approach enables the generation of a smooth surface that can be scaled with a user-defined level of precision, which can be specified during the prototyping stage. In contrast, polygonal models often exhibit varying level of detail across different planes, leading to inconsistencies in surface representation.

### 3.2. Surgical Planning Error Analysis

An analysis of the main errors in planning surgical interventions was conducted. The total positioning error of the surgical instrument ($\delta_{R}$) is a combination of the visualization error ($\delta_{V}$) and the mechanical positioning error of the manipulator ($\delta_{P}$) [40,41,42].

The guidance error is considered, which, in the general case, for root mean square errors $\delta_{1}$ and $\delta_{2}$ with the correlation coefficient could be determined according to the equation:

(27) $$\delta_{\sum }=\sqrt{\delta_{1}^{2}+\delta_{2}^{2}+2K\delta_{1}\delta_{2}}.$$

Given the lack of correlation between errors in determining coordinates (visualization) at the “hemangioma—healthy tissue” boundary and positioning of the (mechanical) surgical instrument, we obtain the value of the resulting root mean square error of the instrument positioning:

(28) $$\delta_{R}=\sqrt{\delta_{V}^{2}+\delta_{P}^{2}}.$$

The visualization error is influenced by factors like imaging resolution and registration accuracy [43,44,45,46]. Given the high correlation of errors from image-based calculations, the hemangioma visualization error is defined as:

(29) $$\delta_{V}=\delta_{T}+\delta_{С}+\delta_{М},$$

where—$\delta_{T}=0.61$ is the error in data representation from radial sections, calculated as:

(30) $$\delta_{T}=\sqrt{\delta_{Tx}^{2}+\delta_{Ty}^{2}+\delta_{Tz}^{2}},$$

and its value is determined basing on the considerations that the spatial resolution errors by coordinates are equal to half the spatial resolution values of the three-dimensional model by the corresponding coordinates, ${x\to \delta }_{Tx}=0.25\;\mathrm{mm},\;{y\to \delta }_{Ty}=0.25\;\mathrm{mm}$, ${z\to \delta }_{Tz}=0.5\;\mathrm{mm}$ (the numbers are indicated for the three-dimensional model used in the work, built based on high-resolution radial cross-section data);

—$\delta_{C}=1.13\;\mathrm{mm}$ is the error in determining the healthy tissue boundary;

—$\delta_{M}=0.5\;\mathrm{mm}$ is the methodological error in defining the coordinates of the surgical area center.

This results in a mean square visualization error of $\delta_{V}=2.24\;\mathrm{mm}$.

Positioning error of the surgical instrument by the manipulator $\delta_{P}$ is determined by the errors $\delta_{P_{1}},\;\delta_{P_{2}},\;\delta_{P_{3}}$ of the mechanical part of the drives in each of the 3 degrees of freedom, respectively:

(31) $$\delta_{P}=\sqrt{\delta_{P_{1}}^{2}+\delta_{P_{2}}^{2}+\delta_{P_{3}}^{2}},$$

and which will consist of the following values: backlash $\delta_{P_{BL*}}$, movement unevenness (tolerance for kinematic error $\delta_{P_{ME*}}$), from shaft twisting $\delta_{P_{ST*}}$, from the supports gap $\delta_{P_{SG*}}$, the clutch $\delta_{P_{C*}}$ and temperature instability $\delta_{P_{T*}}$ according to the following equation:

(32) $$\delta_{P*}=\delta_{P_{BL*}}+\delta_{P_{ME*}}+\delta_{P_{ST*}}+\delta_{P_{SG*}}+\delta_{P_{C*}}+\delta_{P_{T*}},$$

where ∗ denotes the index of each of the 3 degrees of freedom of the surgical instrument in the manipulator.

**Figure 14:**
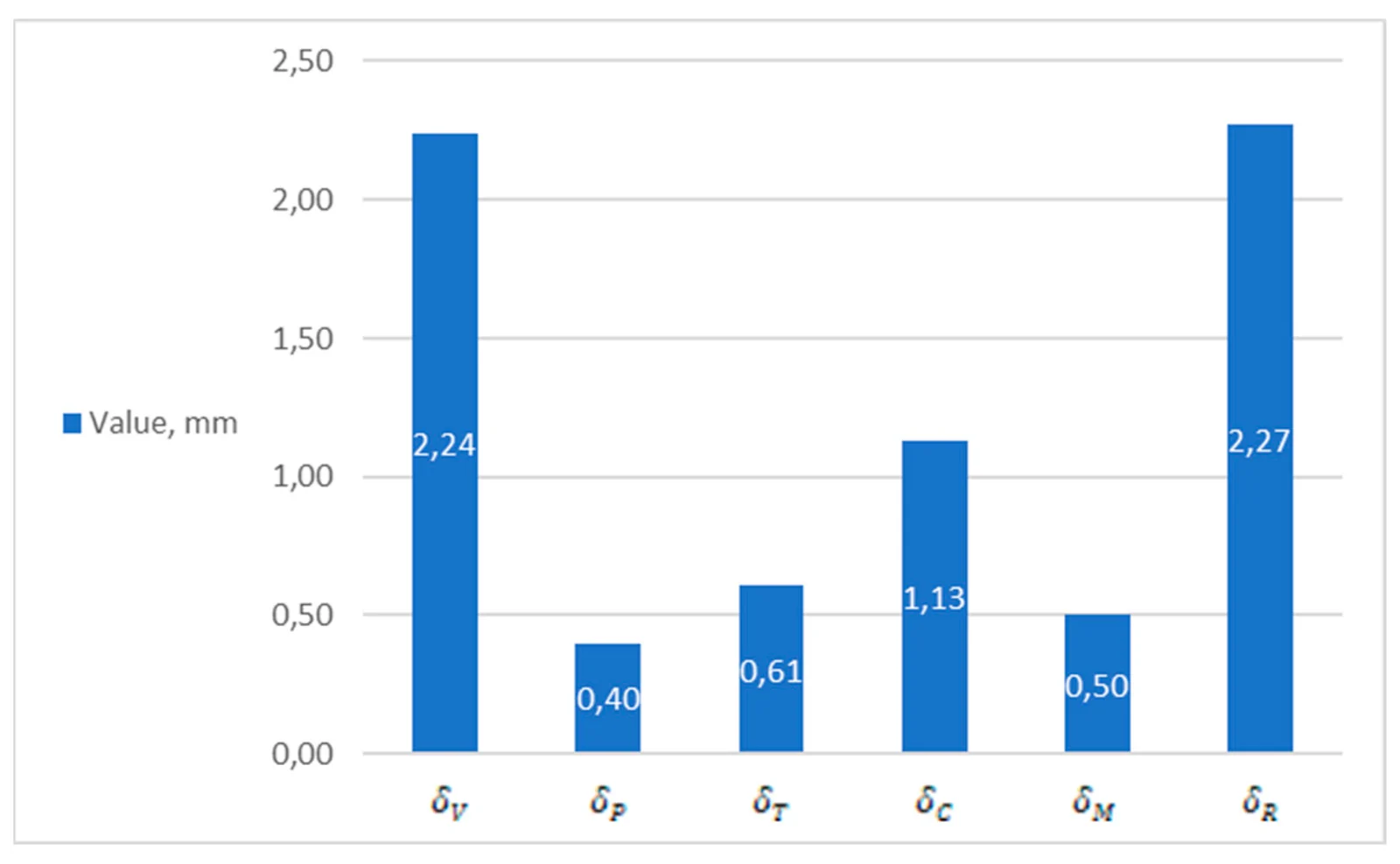
Error values during surgical navigation.

The error values are calculated according to the manuals for mechanical transmissions and they depend on the specific numerical characteristics of the drive design. Based on the above-specified, the error of the positioning of the surgical instrument by the mechanical subsystem of the manipulator is set ($\delta_{P}=0.41\;\mathrm{mm}$). Taking into account these considerations, the resulting error in the positioning of the surgical instrument practically does not increase and equals to 2.27 mm. Accurately estimating and compensating for these errors is a key challenge in computer-assisted surgery [47,48,49]. The largest contributor to this error is the coordinate visualization, which can be reduced by using higher-resolution medical imaging devices and refining calculation methodologies [30], in which the target point will be displayed relative to certain reference landmarks and directly visualized without recalculation algorithms that introduce errors due to the complexity of accounting for the parameters of individual anatomical variability. The obtained error values are given in Figure 14.

**Figure 15:**
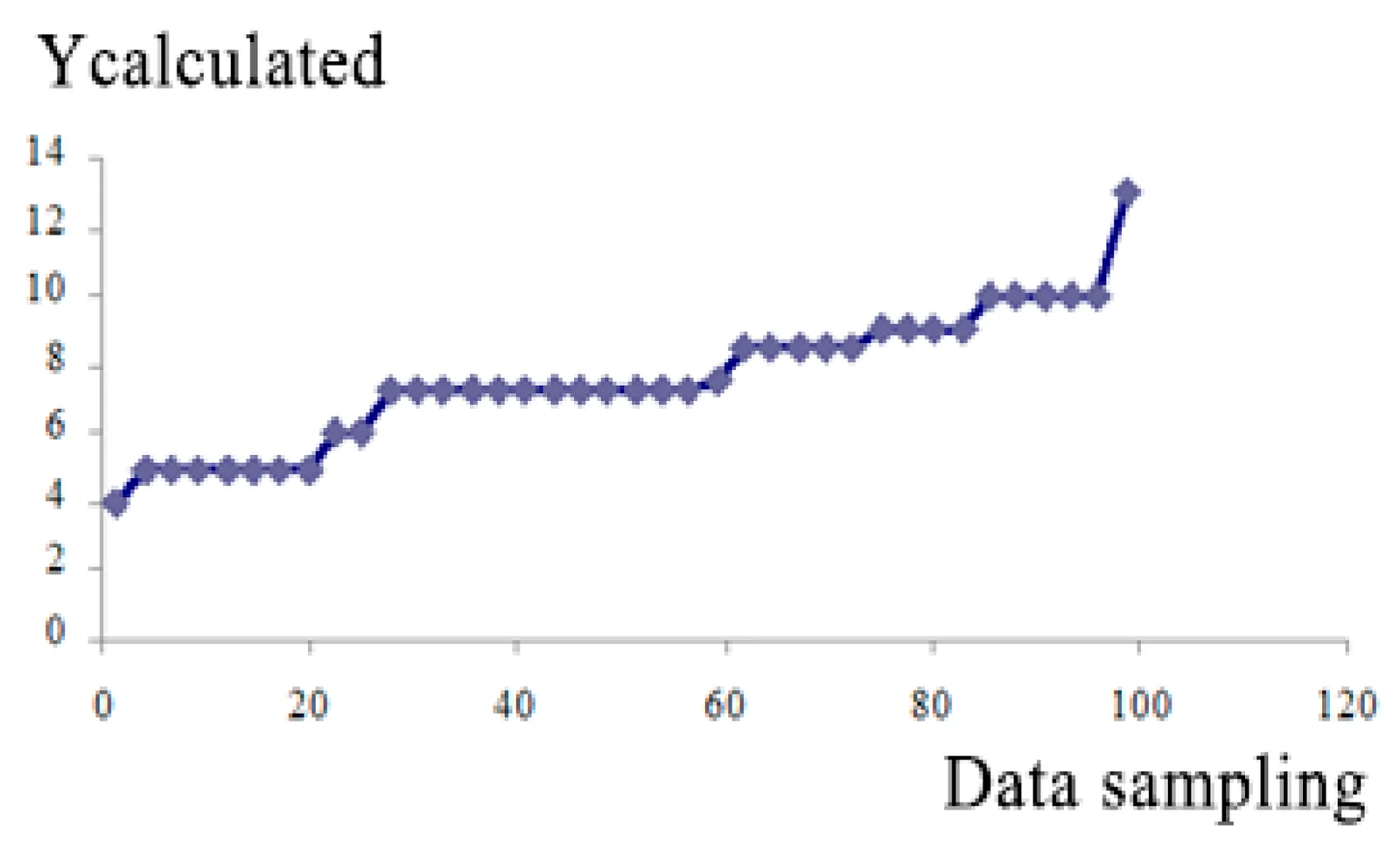
Linear regression equation (Y_calculated_—calculated value of hemangioma resection parameters).

In the fifth data group, the linear regression equation (Figure 15) has the following form:

(33) y=0.751+0.26x.

The standard deviation of the residual series values is: S = 0.579.

Average approximation error: $\bar{A}=5.6\%$.

Since the average approximation error is 5.6%, the quality of the constructed model can be assessed as good, since the average approximation error does not exceed 10%.

**Figure 16:**
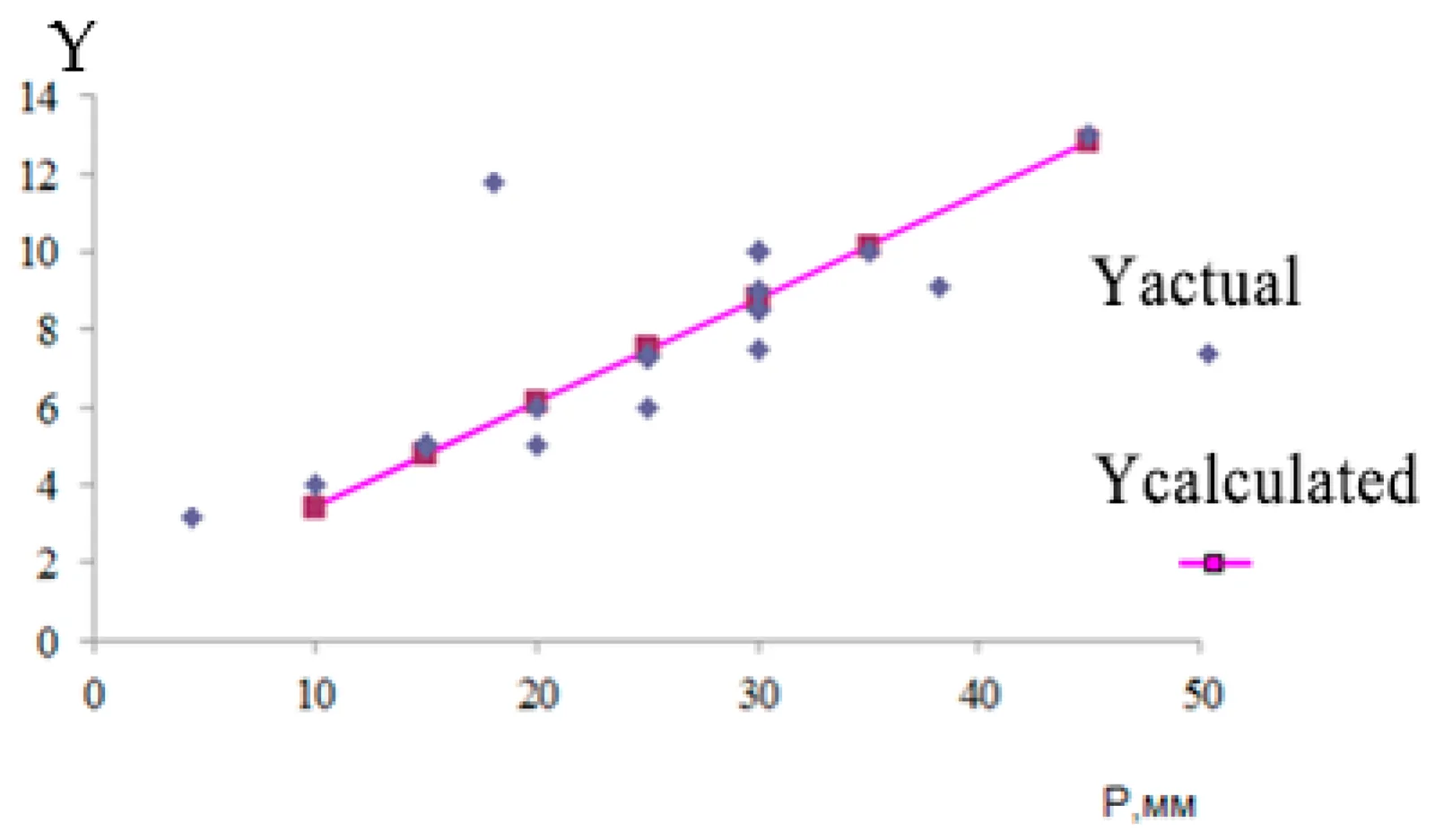
Graph of coincidence of calculated resection parameters with actual ones.

The graph of the coincidence of the calculated resection parameters with the actual ones is shown in Figure 16.

**Figure 17:**
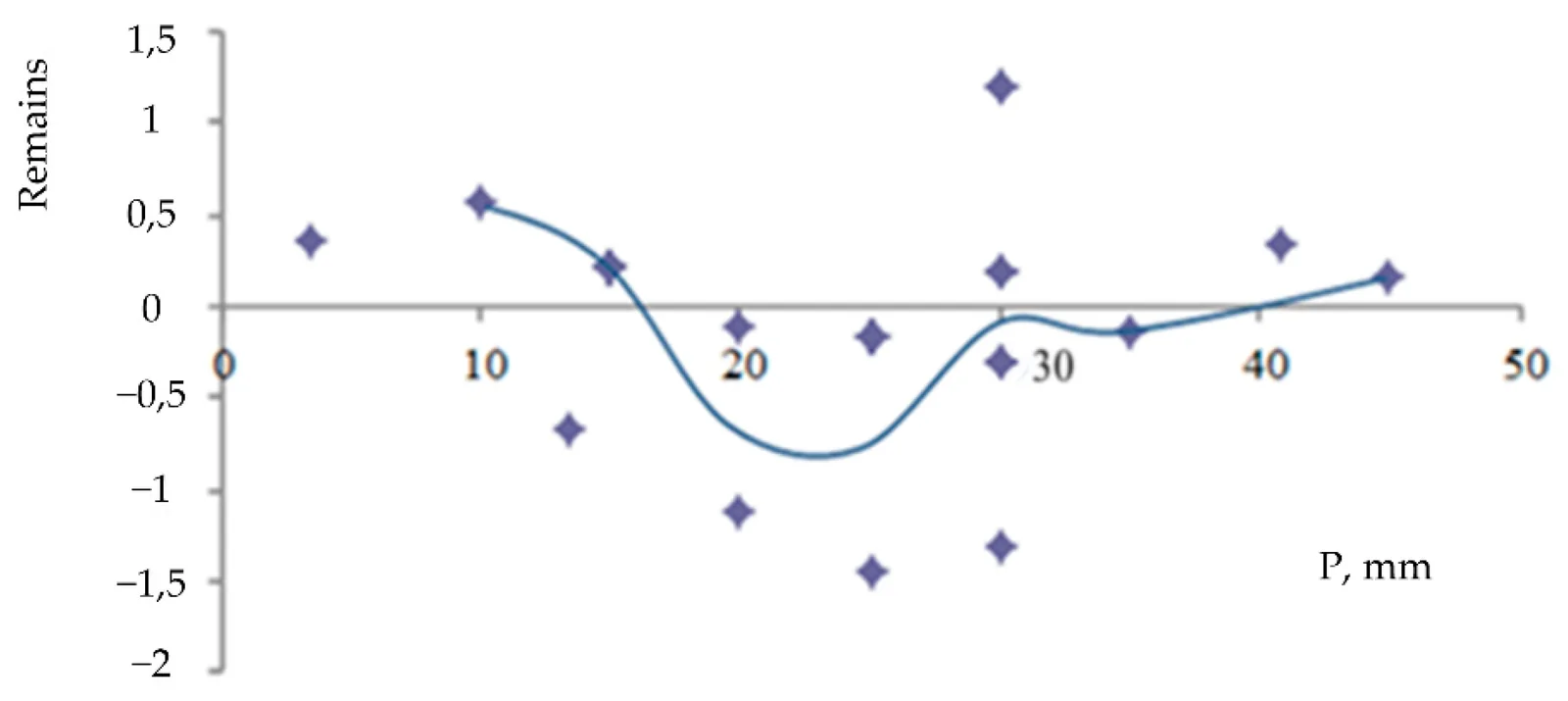
Fisher residual plot.

The adequacy of the linear regression model was further confirmed by an analysis of its residuals. The residual plot (Figure 17), which shows the difference between predicted and actual values, displays a random pattern with no discernible structure, indicating that the model’s assumptions are met. This, along with a high coefficient of determination (R2), confirms the model’s goodness-of-fit.

The model’s quality was assessed as good, with an average approximation error of 5.6%, which is well below the acceptable threshold of 10%.

### 3.3. Discriminant Analysis of Resection Parameters

A discriminant analysis was performed on five parameters influencing the outcome of hemangioma resection: skin elasticity, tumor volume, tumor weight, tumor density and a correction factor (P) for shape and size.

The Mahalanobis distance to compare these parameters in normal versus pathological states, and is calculated using the following equation:

(34) $$\delta =\sqrt{\sum_{i=1}^{n}{\left(\frac{m_{i}^{\left(0\right)}-m_{i}^{\left(1\right)}}{\sigma_{i}}\right)}^{2}}$$

where $m_{i}^{\left(0\right)}$, $m_{i}^{\left(1\right)}$ and $\sigma_{i}^{\left(0\right)}$, $\sigma_{i}^{\left(1\right)}$ are the average value and standard deviation of the corresponding indicators, where $\sigma_{i}=\max\left(\sigma_{i}^{\left(0\right)},\;\sigma_{i}^{\left(1\right)}\right)$. The probability of a decision error will be determined by the following equation:

(35) $$P_{err}\leq 1-\Phi \left(\frac{\delta }{2}\right).$$

Five informative parameters were chosen for calculations. Table 1 shows the results of the calculations of the discriminant characteristics of these five parameters. $\Theta_{0}$ and $\Theta_{1}$ represent the state of the control object in normal and pathological conditions, respectively.

**Figure 18:**
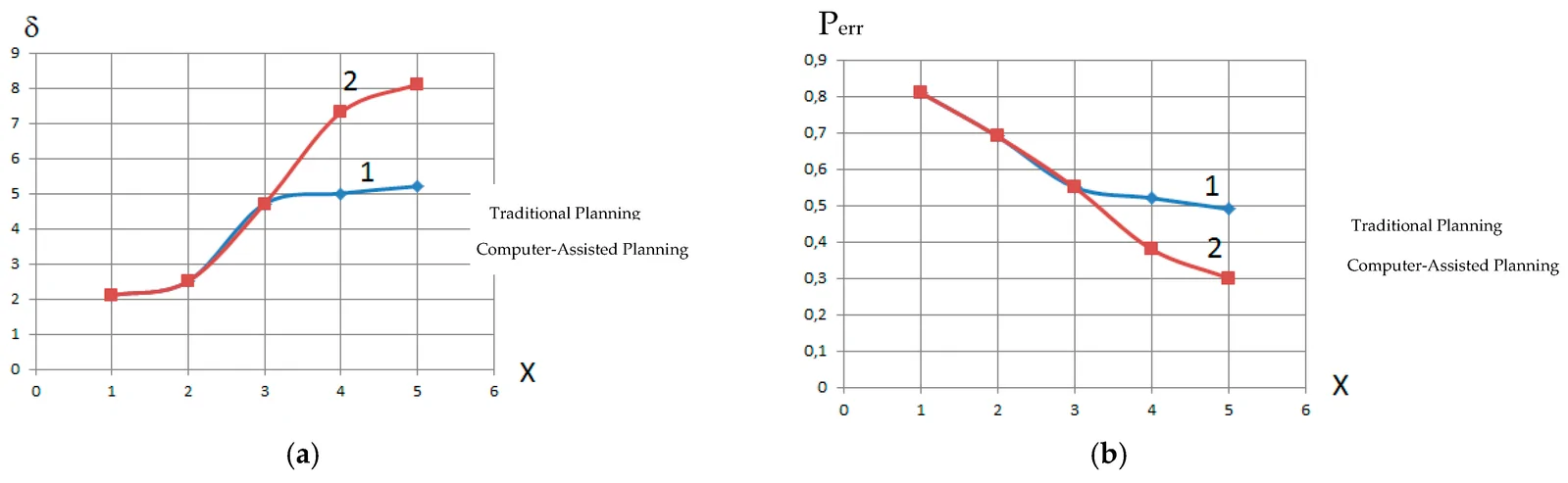
Results of calculations of discriminant characteristics of five influential parameters: (**a**) calculation of Mahalanobis distance; (**b**) calculation of error probability.

The analysis showed that the computer-assisted model which includes additional mathematical coefficients, increased the Mahalanobis distance for tumor density and the P-factor by 2.3 and 2.89, respectively, while reducing the probability of error by 14% compared to traditional assessment. In Figure 18a,b, the curve labeled ‘Traditional Planning’ represents the result of calculating the discriminant characteristics of the five parameters on which the surgeon relied when performing surgical interventions. The curve labeled ‘Computer-Assisted Planning’ represents the result of the calculation based on the parameters used by our preoperative planning system.

Figure 18b demonstrates that all parameters significantly influence the probability of making a diagnostic decision and, therefore, cannot be excluded from the analysis. The practical impact of the dimensionality of the informative parameter space on the probability of preoperative planning errors is also illustrated.

The total time required to apply this methodology for a single patient case, from initial image acquisition to the generation of a final 3D model for preoperative planning, is estimated to be approximately 3 to 4 h. This timeline includes approximately 30 min for standardized photographic image acquisition and system setup, 2 to 3 h for semi-automated image processing and computational modeling, and a final 30 min for surgeon review and interactive planning with the generated 3D model.

### 3.4. Limitations and Future Work

A primary limitation of this study is its scope as a proof-of-concept, demonstrating the feasibility of our proposed method on a limited dataset. We have rigorously validated the computational accuracy of the model, achieving a low average approximation error of 5.6%. However, a large-scale clinical validation study is required to compute robust statistical metrics such as diagnostic accuracy, sensitivity, and specificity. Such a study would involve applying the method to a large cohort of patients and comparing the model-based surgical plans to actual surgical outcomes, which constitutes the next phase of our research.

## 4. Economical Evaluation of the Proposed Method Efficiency

**Figure 19:**
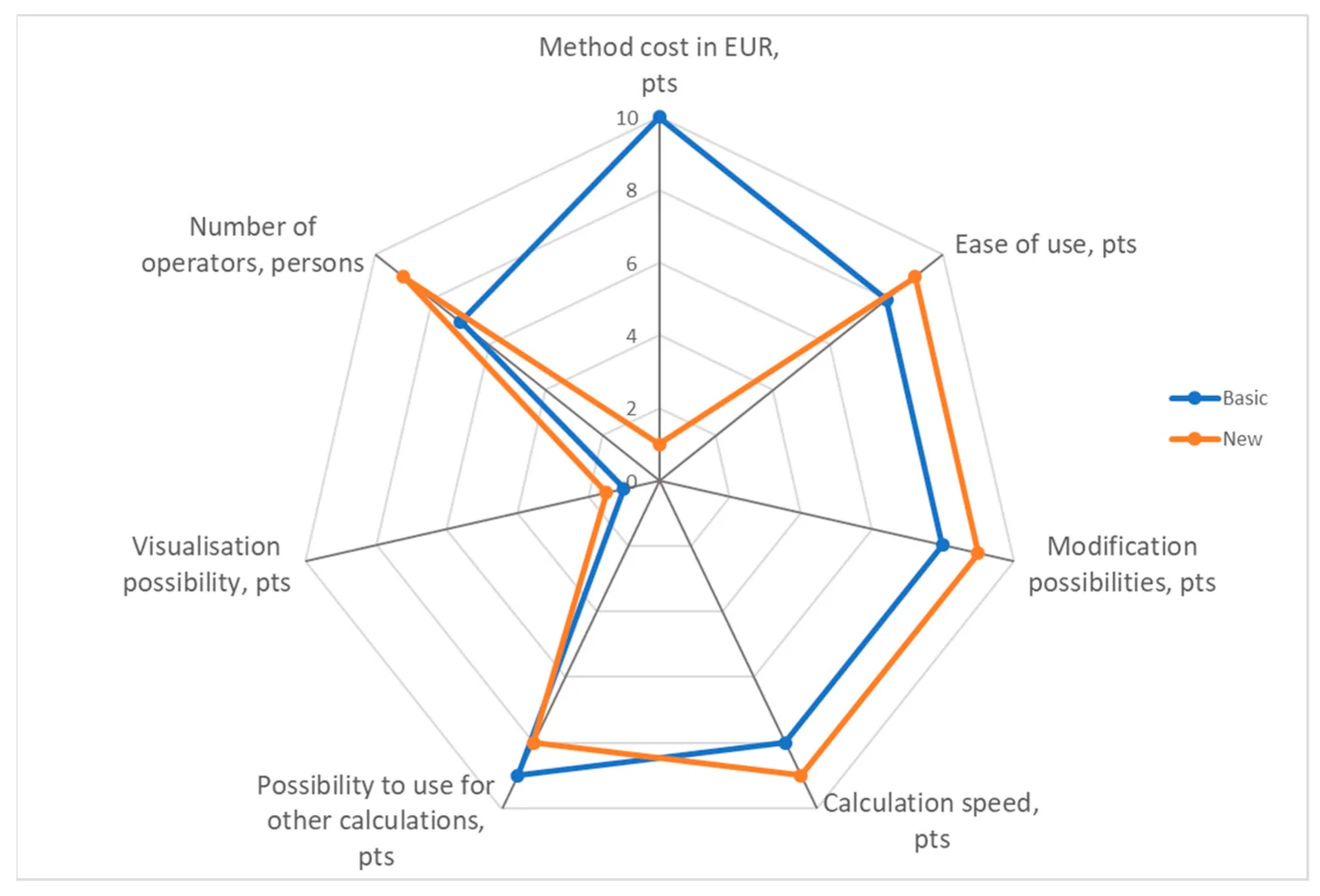
Radar factor chart for comparing competitiveness parameters of the basic and new calculation method options.

An economic analysis was conducted to assess the feasibility of the proposed method. The total one-time development cost for the prototype software and methodology was calculated to be €662.95, with a projected single-license market price of €954.64. This cost was based on the 56-day development and implementation period of the initial project. It is important to note that these figures are not static. The initial development cost is subject to factors such as local labor costs for specialized programmers and the price of required software licenses. However, the cost for deploying the finalized system in additional clinical settings would be substantially lower, primarily consisting of software licensing and user training, making it an economically competitive tool for medical institutions. A comparison of the new method against the basic (existing) method across several metrics is shown in the factor chart (Figure 19).

## 5. Discussion and Conclusions

This research addresses the urgent need for advanced modeling of complex biological objects by presenting a novel method for creating 3D models for preoperative planning of hemangioma surgery. The initial phase of hemangioma is marked by rapid growth, followed by slow and inevitable involution. Current treatment strategies are largely empirical and often unsatisfactory, primary due to limited understanding of the cellular and molecular mechanisms underlying hemangioma development. Progress in elucidating its programmed biological behavior is hindered by the absence of an accurate human model.

Accurate diagnosis and effective treatment require determination of the tumor type, growth characteristics, structure, and anatomical location. Existing methods for surgical planning often rely on generalized “average person” data, which fails to account for individual anatomic variations, making high-quality interventions challenging. The integration of computer-assisted planning technologies can substantially enhance surgical outcomes by expediting the planning process, minimizing the human error, anticipating potential intraoperative complications, and identifying strategies to mitigate such risk. The primary objective of computer-assisted planning is to identify, among numerous possible surgical pathways, the least traumatic approach tailored to the patient’s unique anatomy.

Our use of computer planning technologies can significantly accelerate and simplify the surgeon’s workflow, reduce errors, and improve patient outcomes. We successfully developed a method for generating a 3D surface model from 2D images using polynomial approximation and spline modeling, which is more accurate and scalable than traditional polygonal modeling.

In constructing a 3D qualitative model of hemangioma, spline modeling was chosen over polygonal modeling due to its superior accuracy and consistent quality during scaling. In this method, the hemangioma shape is defined by a series of curves aligned along the equator of a reference sphere. The spline-based surface can be scaled to any desired level of precision, specified during the prototyping stage. In contrast, polygonal models often exhibit inconsistent detail across different planes.

The 3D reconstruction method presented in this paper, based on polynomial and spline interpolation of 2D cross-sections, offers a distinct balance of accessibility, cost, and accuracy for modeling external biological surfaces. It is important to contrast this approach with other established techniques. Volumetric methods using CT or MRI data provide comprehensive 3D models of both surface and internal structures but are associated with high costs, patient radiation exposure (in the case of CT), and may not be readily available. In contrast, our image-based method is low-cost and non-invasive. Compared to other image-based techniques like photogrammetry, which require numerous images from unstructured viewpoints, our approach simplifies the data acquisition and processing by using a structured rotational capture. Furthermore, while active scanning methods like structured light or laser scanning can yield highly accurate surface models, they necessitate specialized hardware, whereas our method can be implemented with a standard digital camera, making it highly accessible for a wide range of clinical settings.

Dedicated software was developed for preoperative planning of surgical interventions on hemangiomas. The software process input data acquired from a medical imaging device to construct a spatial model of the hemangioma. The user can select two arbitrary points on the model to compute the resection dimensions. Based on the reconstructed surface geometry (including protrusions and depressions), the depth of the tumor relative to healthy tissue can be estimated.

Errors in surgical planning were quantitatively evaluated. The primary source of guidance error was found to be visualization error of coordinate points ($\delta_{V}$ = 2.24 mm). This can be significantly reduced by improving the resolution of imaging devices and enhancing the accuracy of computational methods. In particular, ensuring that the target point is visualized relative to anatomical variability.

Discriminant analysis identified five parameters that significantly influence the outcome of hemangioma resection: skin elasticity coefficient, tumor volume, tumor mass, tumor density, and a correction factor P, which accounts for the shape and size of the hemangioma. The adequacy of the proposed three-dimensional spline-based model was validated using Fisher’s and Student’s criteria, demonstrating statistical significance across all five examined groups.

Despite the promising results, we acknowledge several limitations of the current study. First, the accuracy of our 3D reconstruction is highly dependent on the quality of the input 2D images; factors such as inconsistent lighting, low resolution, or patient movement during image capture can introduce errors into the final model. Second, this study serves as a proof-of-concept, and the methodology has been tested on a limited number of cases. Future work should involve a larger clinical study to validate its robustness across a diverse range of hemangioma sizes and shapes. Third, our method models the surface topography and does not provide information on the tumor’s internal vasculature or depth of tissue invasion, which must be assessed using complementary imaging modalities like MRI or ultrasound. Finally, while polynomial and spline modeling are effective, future research could explore alternative 3D reconstruction techniques, such as photogrammetry or structured light scanning, which may offer improved accuracy or a more streamlined workflow.

This works provides a robust, accurate and economically viable solution to enhance the precision of hemangioma surgery, contributing to the ongoing evolution of the “operating room of the future”.

## Figures and Tables

**Table 1:** Results of calculations of discriminant characteristics of five parameters. The Mahalanobis distance is a unitless metric as it is normalized by the standard deviation and covariance of the data.

Parameter		Condition of the Control Object		Mahalanobis Distance δ	Probability of Error $P_{err}$
		$\Theta_{0}$	$\Theta_{1}$		
$X_{1}$	Skin elasticity coefficient	1.1	0.8	2.1	≤0.81
$X_{2}$	Tumor volume, mm^3^	4	1	2.5	≤0.69
$X_{3}$	Tumor weight, g	0	17.5	6.40	≤0.45
$X_{4}$	Tumor density, g/mm^3^	11	9	7.3	≤0.4
$X_{5}$	P- correction factor that specifies the shape and size of the hemangioma	0.67	0.97	8.11	≤0.31

## Data Availability

The original contributions presented in this study are included in the article. Further inquiries can be directed to the corresponding author.

## References

[1] Long D.M. (1999). The operating room of the future. Neurol. Res..

[2] Tessman C. (1999). Exploring frameless stereotactic image guided surgery. AORN J..

[3] Vannier M.W., Haller J.W. (1999). Navigation in diagnosis and therapy. Eur. J. Radiol..

[4] Hassfeld S., Zöller J., Albert F.K., Wirtz C.R., Knauth M., Mühling J. (1998). Preoperative planning and intraoperative navigation in skull base surgery. J. Cranio-Maxillofac. Surg..

[5] Very M., Nagy M., Carr M., Collins S., Brodsky L. (2002). Hemangiomas and vascular malformations: Analysis of diagnostic accuracy. Laryngoscope.

[6] Kowalska M., Dębek W., Matuszczak E. (2021). Infantile Hemangiomas: An Update on Pathogenesis and Treatment. J. Clin. Med..

[7] Butnor K.J., Khoor A. (2008). Collagen Vascular Diseases and Disorders of Connective Tissue. Dail and Hammar’s Pulmonary Pathology: Volume I: Nonneoplastic Lung Disease.

[8] Puxeddu R., Berlucchi M., Ledda G.P., Parodo G., Farina D., Nicolai P. (2006). Lobular capillary hemangioma of the nasal cavity: A retrospective study on 40 patients. Am. J. Rhinol..

[9] Rešić A., Barčot Z., Habek D., Pogorelić Z., Bašković M. (2025). The Evaluation, Diagnosis, and Management of Infantile Hemangiomas—A Comprehensive Review. J. Clin. Med..

[10] Yurko O.O., Tikhomirova D.V., Kukharenko D.V. Obtaining the analytical dependence of a three-dimensional surface. Proceedings of the Production & Mechatronic Systems IV International Conference.

[11] Yurko O.O., Tikhomirova D.V., Kukharenko D.V., Prohorova-Kim M. Simulation of the volume surface according to the radial sections of the figure. Proceedings of the 19th International Conference on Physical Processes and Fields of Technical and Biological Objects.

[12] Baumhauer M., Feuerstein M., Meinzer H.P., Rassweiler J. (2008). Navigation in endoscopic soft tissue surgery: Perspectives and limitations. J. Endourol..

[13] Bobek S.L. (2014). Applications of navigation for orthognathic surgery. Oral Maxillofac. Surg. Clin..

[14] Keschner D., Lee J. (2010). Use of surgical navigation during endoscopic skull base surgery. Oper. Tech. Otolaryngol.-Head Neck Surg..

[15] Pagliai K.A., Cohen B.A. (2004). Pyogenic granuloma in children. Pediatr. Dermatol..

[16] Pascual-Castroviejo I., Pascual-Pascual S.I., López-Gutiérrez J.C., Velazquez-Fragua R., Viaño J. (2007). Facial hemangioma and hemispheric migration disorder: Presentation of 5 patients. Am. J. Neuroradiol..

[17] Langdon C., Hinojosa-Bernal J., Munuera J., Gomez-Chiari M., Haag O., Veneri A., Valldeperes A., Valls A., Adell N., Santamaria V. (2023). 3D printing as surgical planning and training in pediatric endoscopic skull base surgery—Systematic review and practical example. Int. J. Pediatr. Otorhinolaryngol..

[18] Zoabi A., Redenski I., Oren D., Kasem A., Zigron A., Daoud S., Moskovich L., Kablan F., Srouji S. (2022). 3D Printing and Virtual Surgical Planning in Oral and Maxillofacial Surgery. J. Clin. Med..

[19] Ford J.M., Decker S.J. (2016). Computed tomography slice thickness and its effects on three-dimensional reconstruction of anatomical structures. J. Forensic Radiol. Imaging.

[20] Ettarh R. (2014). Pocket Atlas of Sectional Anatomy: Computed Tomography and Magnetic Resonance Imaging, Volume I: Head and Neck; Volume II: Thorax, Heart, Abdomen, and Pelvis. J. Anat..

[21] Macca L., Altavilla D., Di Bartolomeo L., Irrera N., Borgia F., Li Pomi F., Vaccaro F., Squadrito V., Squadrito F., Vaccaro M. (2022). Update on Treatment of Infantile Hemangiomas: What’s New in the Last Five Years?. Front. Pharmacol..

[22] Valverde I., Gomez G., Coserria J.F., Suarez-Mejias C., Uribe S., Sotelo J., Velasco M., Santos De Soto J., Hosseinpour A.R., Gomez-Cia T. (2015). 3D printed models for planning endovascular stenting in transverse aortic arch hypoplasia. Catheter. Cardiovasc. Interv..

[23] Gao Y., Jiang Y., Peng Y., Yuan F., Zhang X., Wang J. (2025). Medical Image Segmentation: A Comprehensive Review of Deep Learning-Based Methods. Tomography.

[24] Meyer-Szary J., Luis M.S., Mikulski S., Patel A., Schulz F., Tretiakow D., Fercho J., Jaguszewska K., Frankiewicz M., Pawłowska E. (2022). The Role of 3D Printing in Planning Complex Medical Procedures and Training of Medical Professionals—Cross-Sectional Multispecialty Review. Int. J. Environ. Res. Public Health.

[25] Yan Q., Dong H., Su J., Han J., Song B., Wei Q., Shi Y. (2018). A review of 3D printing technology for medical applications. Engineering.

[26] Mithun N.C., Howlader T., Rahman S.M. (2016). Video-based tracking of vehicles using multiple time-spatial images. Expert Syst. Appl..

[27] Yang J., Yuan J., Li Y. (2016). Parsing 3D motion trajectory for gesture recognition. J. Vis. Commun. Image Represent..

[28] Sommer O., Dietz A., Westermann R., Ertl T. (1999). An interactive visualization and navigation tool for medical volume data. Comput. Graph..

[29] Chau W., McIntosh A.R. (2005). The Talairach coordinate of a point in the MNI space: How to interpret it. Neuroimage.

[30] Lancaster J.L., Tordesillas-Gutiérrez D., Martinez M., Salinas F., Evans A., Zilles K., Mazziotta J.C., Fox P.T. (2007). Bias between MNI and Talairach coordinates analyzed using the ICBM-152 brain template. Hum. Brain Mapp..

[31] Laird A.R., Robinson J.L., McMillan K.M., Tordesillas-Gutiérrez D., Moran S.T., Gonzales S.M., Ray K.L., Franklin C., Glahn D.C., Fox P.T. (2010). Comparison of the disparity between Talairach and MNI coordinates in functional neuroimaging data: Validation of the Lancaster transform. Neuroimage.

[32] Yang J., Wang Y., Liu Y., Tang S., Chen W. (2009). Novel approach for 3-d reconstruction of coronary arteries from two uncalibrated angiographic images. IEEE Trans. Image Process..

[33] Janssen R., Verrijt M., de Best J., van de Molengraft R. (2012). Ball localization and tracking in a highly dynamic table soccer environment. Mechatronics.

[34] Lin Y.H., Wu W.H., Huang W.Z. (2011). High speed 3D motion capture system for flying golf ball. Phys. Procedia.

[35] Inal M., Muluk N.B., Burulday V., Akgül M.H., Ozveren M.F., Çelebi U.O., Şimşek G., Daphan B.Ü. (2016). Investigation of the calcification at the petroclival region through multi-slice computed tomography of the skull base. J. Cranio-Maxillofac. Surg..

[36] Appelbaum L., Sosna J., Nissenbaum Y., Benshtein A., Goldberg S.N. (2011). Electromagnetic navigation system for CT-guided biopsy of small lesions. Am. J. Roentgenol..

[37] Christie S. (2014). Electromagnetic navigational bronchoscopy and robotic-assisted thoracic surgery. AORN J..

[38] Hall W., Liu H., Martin A., Truwit C. (2000). Intraoperative magnetic resonance imaging. Top. Magn. Reson. Imaging.

[39] Sidhu R., Weir-McCall J., Cochennec F., Riga C., DiMarco A., Bicknell C.D. (2012). Evaluation of an electromagnetic 3D navigation system to facilitate endovascular tasks: A feasibility study. Eur. J. Vasc. Endovasc. Surg..

[40] Bruners P., Penzkofer T., Nagel M., Elfring R., Gronloh N., Schmitz-Rode T., Günther R.W., Mahnken A.H. (2009). Electromagnetic tracking for CT-guided spine interventions: Phantom, ex-vivo and in-vivo results. Eur. Radiol..

[41] Stathopoulos I., Karampinas P., Evangelopoulos D.S., Lampropoulou-Adamidou K., Vlamis J. (2013). Radiation-free distal locking of intramedullary nails: Evaluation of a new electromagnetic computer-assisted guidance system. Injury.

[42] Sagi H.C., Manos R., Benz R., Ordway N.R., Connolly P.J. (2003). Electromagnetic field-based image-guided spine surgery part one: Results of a cadaveric study evaluating lumbar pedicle screw placement. Spine.

[43] Bandettini P., Laurentius H., Finn E. (2021). Challenges and opportunities of mesoscopic brain mapping with fMRI. Curr. Opin. Behav. Sci..

[44] Fazio P., Schain M., Varnäs K., Halldin K., Farde L., Varrone A. (2016). Mapping the distribution of serotonin transporter in the human brainstem with high-resolution PET: Validation using postmortem autoradiography data. NeuroImage.

[45] Nagaratnam N., Nagaratnam K. (2016). Enlargement of the posterior horns of the lateral ventricles and recurrent falls: A clinical study. J. Clin. Gerontol. Geriatr..

[46] Nowinski W.L., Chua B.C., Volkau I., Puspitasari F., Marchenko Y., Runge V.M., Knopp M.V. (2010). Simulation and assessment of cerebrovascular damage in deep brain stimulation using a stereotactic atlas of vasculature and structure derived from multiple 3- and 7-tesla scans. J. Neurosurg..

[47] Nafis C., Jensen V., Beauregard L., Anderson P. Method for Estimating Dynamic EM Tracking Accuracy of Surgical Navigation Tools. Proceedings of the SPIE Medical Imaging Symposium (Medical Imaging 2006).

[48] Nafis C., Jensen V., von Jako R. Method for evaluating compatibility of commercial Electromagnetic. Proceedings of the SPIE Medical Imaging Symposium (Medical Imaging 2006).

[49] Wu J., Hui W., Chen S., Niu J., Lin Y., Luan N., Zhang S., Shen S.G.F. (2020). Error Analysis of Robot-Assisted Orthognathic Surgery. J. Craniofacial Surg..

